# Cascade‐Targeted Nanoparticles for Enhanced Gemcitabine Delivery and Adenosine Metabolism Modulation to Overcome Treatment Resistance in Pancreatic Cancer

**DOI:** 10.1002/advs.202507118

**Published:** 2025-07-08

**Authors:** Hongrui Fan, Hongyi Chen, Haolin Song, Chufeng Li, Yu Wang, Zhenhao Zhao, Qin Guo, Xuwen Li, Mingxuan Liu, Tao Sun, Chen Jiang

**Affiliations:** ^1^ Department of Pharmaceutics School of Pharmaceutical Sciences Key Laboratory of Smart Drug Delivery Ministry of Education State Key Laboratory of Brain Function and Disorders and MOE Frontiers Center for Brain Science Fudan University Shanghai 201203 China

**Keywords:** adenosine metabolism, albumin‐nanoparticle conjugate, drug‐loaded albumin, pancreatic cancer, Therapeutic resistance

## Abstract

KRAS mutations are find in over 90% of pancreatic ductal adenocarcinoma (PDAC) cases, making PDAC exhibit intrinsic resistance to chemotherapy and reshape the immunosuppressive tumor microenvironment (TME), disappointing the clinically preferred chemotherapy‐immunotherapy combination. Standing on the cross point of therapeutic resistance, the aberrant adenosine metabolism contributes greatly to chemo‐ and immunotherapy tolerance. KRAS mutation‐induced over‐expression of key enzyme CD39 is believed to be involved in shaping the immunosuppressive TME, as it catalyzes the hydrolysis of extracellular ATP into immunosuppressive adenosine. Meanwhile, the loss of equilibrative nucleoside transporters (ENTs) leads to the accumulation of adenosine and the intracellular delivery difficulty of gemcitabine, further vanishing patients’ hope of benefiting from either chemotherapy or immunotherapy. The key challenge is to modulate the aberrant metabolism, also enhance gemcitabine intracellular delivery. Therefore, ROS‐responsive positively‐charged polymer B‐PDEA is prepared and assembled into polyplexes for loading CD39‐down‐regulating small interfering RNA. Gemcitabine‐loaded albumin is coupled with the polyplexes through enzyme‐cleavable peptide, forming the intact nanoparticles for the co‐delivery of the first‐line chemotherapeutic drug and CD39‐regulating nucleic acid, showing enhanced gemcitabine intracellular delivery and adenosine metabolism regulating capacity. This approach activated antitumor immunity while achieving chemosensitization by changing the metabolic‐immune crosstalk of TME, showcasing great potential for PDAC treatment.

## Graphical abstract

The aberrant adenosine metabolism leads to chemoresistance and immunosuppressive microenvironment. A versatile albumin‐polyplexes conjugated nanoparticle B‐PDEA@CPA is prepare for the codelivery of gemcitabine and siCD39. The nanoparticle releases gemcitabine‐loaded albumin upon microenvironment to achieve promoted penetration and cellular uptake, while siCD39 can be specifically released to modulate the aberrant metabolism. The nanoparticles promote the chemotherapy and antitumor immune response synergistically.

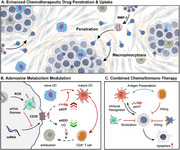

## 1 Introduction

Clinically, over 90% of pancreatic ductal adenocarcinoma (PDAC) cases carry KRAS gene mutations, featuring high invasiveness and malignant proliferation.^[^ ^1^, ^2^, ^3^, ^4^ ^]^ KRAS mutations change the substance transport and metabolic patterns of PDAC,^[^ ^5^, ^6^, ^7^ ^]^ leading to intrinsic resistance to chemotherapy and immunotherapy.^[^ ^8^, ^9^, ^10^ ^]^ Although the first‐line clinical treatment regimen is chemotherapy (gemcitabine + albumin‐bound paclitaxel) + immunotherapy (anti‐PD‐1 therapy, such as pembrolizumab),^[^ ^11^, ^12^ ^]^ the efficacy of the abovementioned combination regimen is still difficult to be satisfactory.

KRAS mutations endow PDAC with selective substance transport ability, eliminating external pressure and shaping an advantageous chemical microenvironment. On the one hand, PDAC forms an inherent barrier to limit the accumulation of adverse factors. KRAS mutations induce the formation of dense extracellular matrix (ECM), passively limiting the perfusion of drugs deep into tumor tissues.^[^ ^11^, ^13^ ^]^ Meanwhile, as the first‐line chemotherapy drug gemcitabine needs to be actively taken up through equilibrative nucleoside transporters (ENTs),^[^ ^14^ ^]^ KRAS mutant diminishes the expression of ENTs, actively limiting the uptake of gemcitabine.^[^ ^15^, ^16^ ^]^ On the other hand, due to the high nutritional requirements, PDAC has evolved an “ admittance” mechanism for nutrients on the basis of maintaining barriers to chemotherapeutic drugs. Through the enhanced macropinocytosis pathway, PDAC cells non‐selectively internalize and catabolize extracellular nutrients represented by albumin to maximize the use of limited resources;^[^ ^17^, ^18^ ^]^ highly expressed matrix metalloproteinases (MMPs) promote angiogenesis and epithelial‐mesenchymal transition (EMT), providing PDAC with continuous and reliable nutritional input and sufficient living space.^[^ ^13^, ^19^ ^]^

More severely, PDAC with KRAS mutations shapes a harsh immune microenvironment through aberrant metabolic pathways to resist the threat of antitumor immunity.^[^ ^20^, ^21^, ^22^ ^]^ Aberrant adenosine metabolism is one of the most typical metabolic reprogramming pathways in PDAC.^[^ ^23^, ^24^ ^]^ Normally, extracellular ATP (eATP) induced by chemotherapy serves as an important “find me” signal and damage‐associated molecular patterns (DAMPs) to promote local immune stimulation and synergistical tumor inhibition.^[^ ^25^, ^26^ ^]^ However, overexpressed extracellular nuclease CD39 in PDAC rapidly hydrolyzes eATP into an immunosuppressive metabolite, extracellular adenosine (eADO), with the assistance of CD73.^[^ ^23^, ^27^, ^28^, ^29^ ^]^ Down‐regulation of ENTs expression in PDAC makes it difficult to remove eADO from tumor microenvironment (TME), resulting in eADO accumulation and promoting PDAC immune escape.^[^ ^26^, ^30^, ^31^, ^32^ ^]^ High expression of metabolic immune checkpoint CD39 and the loss of ENTs jointly participate in PDAC chemo‐ and immunotherapy resistance, making the chemotherapy‐immune cascade unable to be effectively activated.

Therefore, considering the aberrant substance transport and metabolic capabilities of PDAC, utilizing the special “admittance” mechanism for nutrients and thus bypassing the intrinsic barriers could be a feasible strategy for chemotherapeutic drug delivery. Also, reversing the unique metabolic‐immune crosstalk characteristics derived during tumorigenesis by targeting CD39, the key enzyme in the adenosine metabolic axis, is expected to achieve chemosensitization and restore antitumor immune activity, synergistically producing a critical blow to PDAC.

Pharmaceutical methods, especially nanocarriers, provide possibility for changing intracellular drug delivery pathways.^[^ ^33^, ^34^, ^35^ ^]^ As a successful commercial drug carrier, albumin is widely used in antitumor drug delivery due to its capability of tumor targeting and entering tumor cells in large quantities through the transporter‐independent macropinocytosis pathway.^[^ ^36^, ^37^ ^]^ Furthermore, studies have shown that ultra‐small nanoparticles exhibit better tumor penetration without damaging the ECM.^[^ ^38^, ^39^ ^]^ However, particles with ultra‐small sizes often suffer from poor thermodynamic and systemic circulation stability. One of the feasible strategies is to develop a size‐variable drug delivery system.^[^ ^40^, ^41^, ^42^ ^]^ This system will maintain a relatively large size in the circulation to achieve prolonged circulation and reduce in size under the triggering of TME characteristic factors to enhance penetration and overcome the intrinsic treatment resistance.

**Scheme 1:**
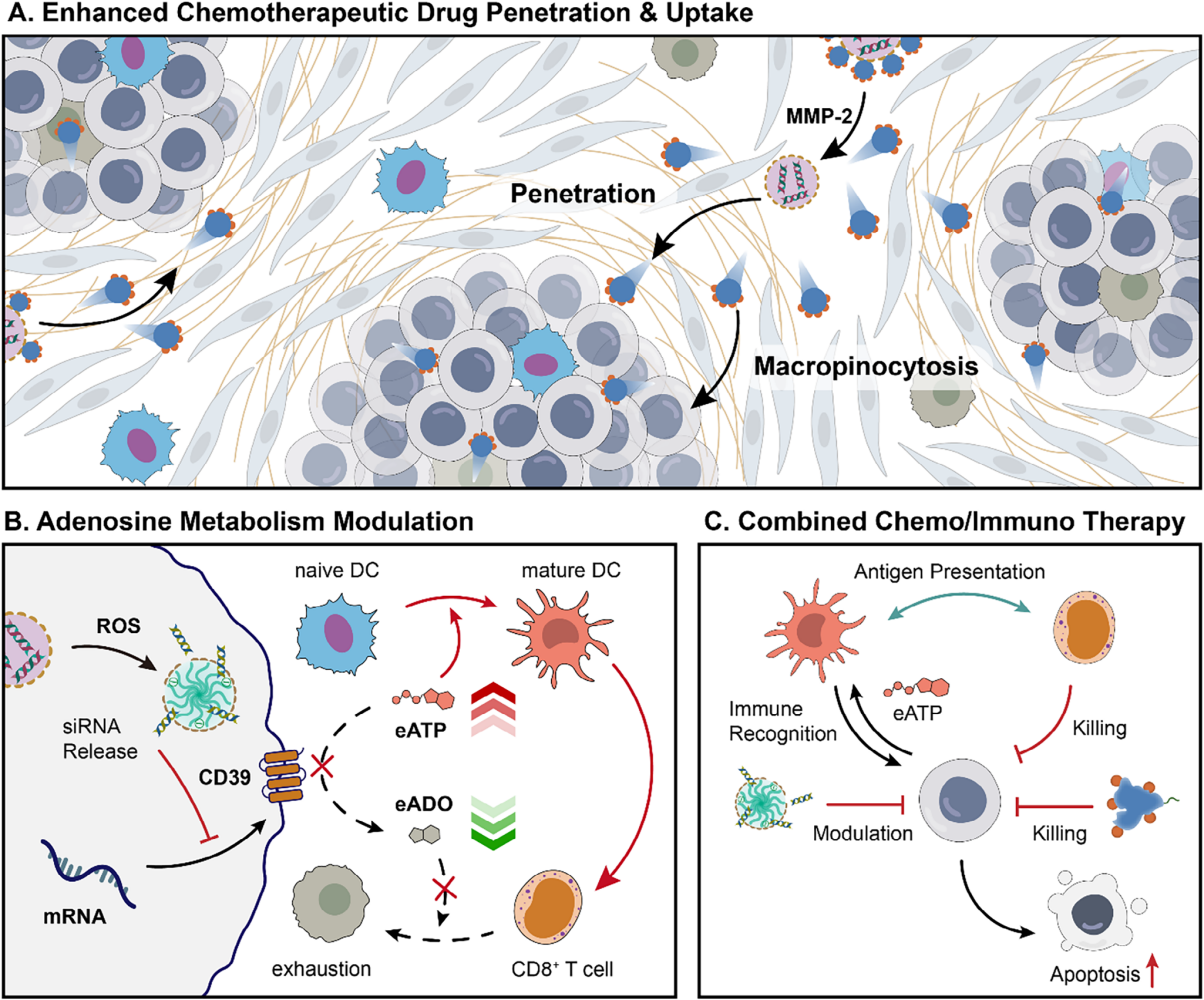
Schematic illustration of the albumin‐based tumor penetration promotion and the enhanced cellular uptake through macropinocytosis. ROS‐triggered siRNA release effectively inhibits the expression of CD39 and adenosine metabolism for the activation of antitumor responses.

Herein, considering the treatment resistance dilemma of PDAC, this study designed and fabricated a drug delivery system for enhanced chemotherapeutic drug delivery and immune activation (**Scheme** **1**). To solve the problem of gemcitabine's intracellular delivery, albumin was chosen as the carrier of gemcitabine, giving full play to its ultra‐small size‐mediated enhanced tumor penetration capacity and macropinocytosis‐mediated enhanced cellular uptake ability, thus improving gemcitabine internalization in an ENT‐independent way. Further, in order to achieve controlled delivery, release and effective transfection of CD39‐regulating small interfering RNA (siCD39), ROS‐responsive charge‐reversible polymer B‐PDEA was used as the delivery carrier, forming drug‐loaded polyplexes with siCD39 via electrostatic interaction‐mediated self‐assembly. MMP‐responsive peptide was used to couple gemcitabine‐loaded albumin and siCD39‐loaded B‐PDEA polyplexes to form the intact nanoparticle with a larger size to improve circulation stability and intratumoral accumulation ability. Due to the strong endogenous demand for albumin in PDAC, albumin‐coupled nanoparticles can be targeted to the tumor lesion and achieve accumulation. Cleaved by local high concentration of MMP‐2 in the TME, smaller gemcitabine‐loaded albumin could be released to realize deeper tumor penetration and enhanced gemcitabine uptake. When siCD39‐loaded polyplexes recognize high‐leveled ROS in PDAC cells, the polymer B‐PDEA achieved charge flipping and selectively released siCD39 to inhibit CD39 expression. In vitro and in vivo experiments have shown that this strategy effectively and synergistically achieves chemotherapy sensitization, metabolic regulation, and immune activation, offering a novel approach and possibility for the treatment of PDAC.

## 2 Results and Discussion

### 2.1 The Preparation and Characterization of the Cascade‐Targeted Nanoparticle B‐PDEA@CPA

Albumin, as one of the most successful commercial nanocarriers, can magnificently promote the delivery of chemotherapeutic drugs and boost their antitumor activity.^[^ ^43^ ^]^ The favorable biocompatibility of albumin leads to prolonged circulation, and its small size allows it to penetrate deep into solid tumors, making it suitable for delivery needs in PDAC treatment. However, the common drug‐loading technique of albumin involves its reversible binding with poorly soluble drugs,^[^ ^36^ ^]^ which is not suitable for the loading and delivery of highly hydrophilic drugs like gemcitabine, the first‐line chemotherapeutic drug for PDAC treatment. To address this issue, this study modified the structure of gemcitabine and synthesized the prodrug comprising a maleimide group, enabling the drug loading through chemical conjugation (Scheme S1, Supporting Information).

The charge‐reversible gene‐drug carrier B‐PDEA was synthesized by reversible addition‐fragmentation chain transfer (RAFT) polymerization and further modified with 4‐(bromomethyl) phenylboronic acid to obtain ROS‐responsive ability (Schemes S2 and S3, Supporting Information). To achieve the responsive dissociation and drug release, as well as to regulate the surface electrical features of the nanoparticles, the B‐PDEA polymers were end‐capped with an MMP‐cleavable peptide‐N‐hydroxysuccinimide^[^ ^44^, ^45^ ^]^ or a carboxyl group, respectively. The chemical structures and degrees of polymerization of all prodrugs, monomers, and polymers were characterized by ^1^H‐NMR (Figures S1–S6, Supporting Information).

**Figure 1:**
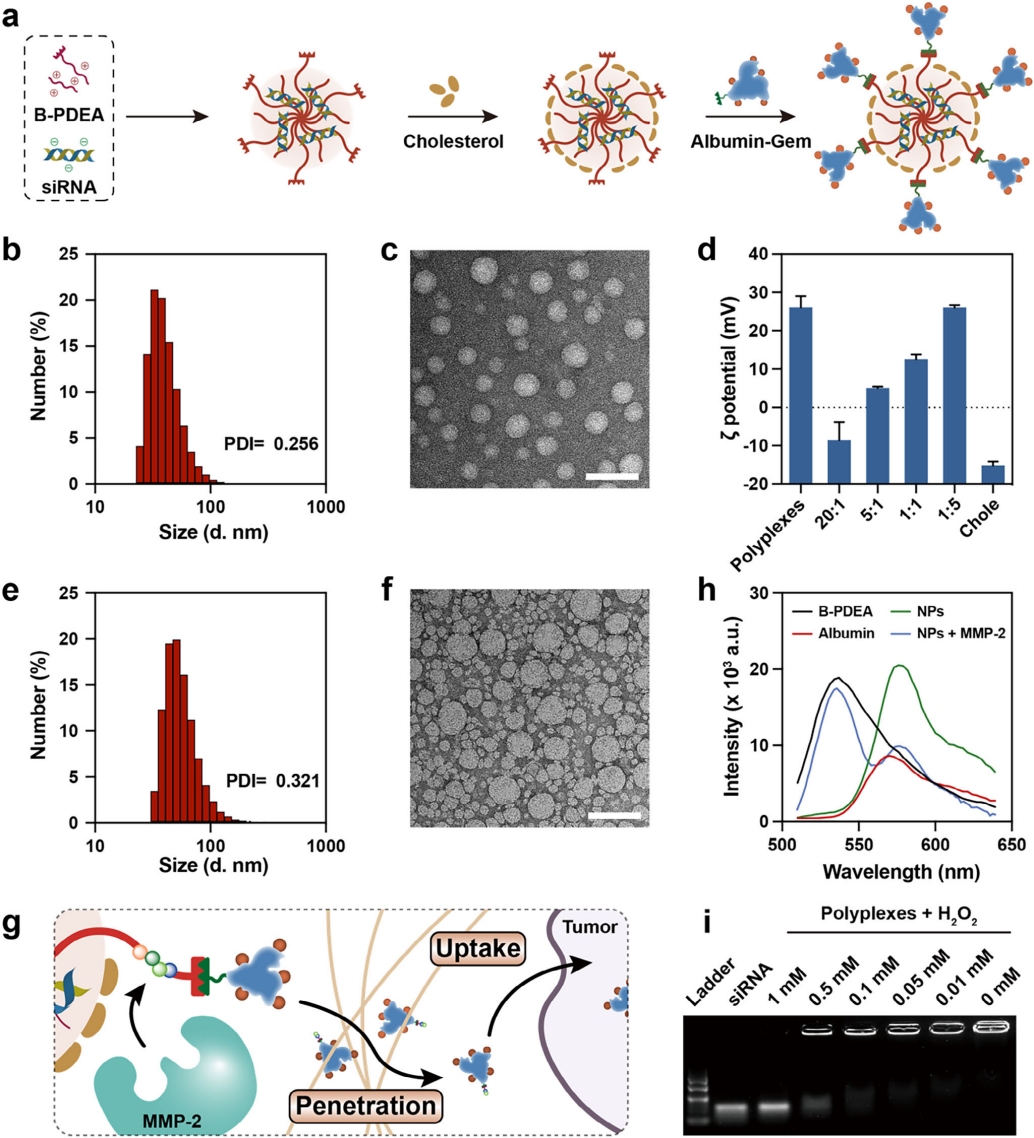
Preparation and characterization of B‐PDEA@CPA. a) Schematic illustration of the preparation and modification procedure of B‐PDEA@CPA. b) Representative size distribution and c) TEM image of B‐PDEA/siCD39 polyplexes at N:P = 15. Scale bar = 100 nm. d) ζ potentials at different polyplexes/cholesterol ratios. e) Representative size distribution and f) TEM image of B‐PDEA@CPA. Scale bar = 100 nm. g) Schematic illustration of the MMP‐2 triggered Albumin‐Gem release, penetration, and enhanced tumor uptake. h) Emission spectra of B‐PDEA‐FITC, Albumin‐Cy3, B‐PDEA@CPA nanoparticles and MMP‐2 treated nanoparticles. λ Exc = 488 nm. i) Gel retardation assay of polyplexes after a 1 h incubation with different concentrations of H_2_O_2_ at 37 °C. The data are represented as the means ± SD (n = 3). Prism software package (PRISM 7.0; GraphPad Software, 2016) was applied for all statistical analyses.

The nanoparticles were prepared according to the process shown in **Figure** **1**a. The siRNA loading capacity of B‐PDEA was explored. After comprehensively evaluating the loading capacity and loading efficiency for siRNA, an N/P ratio of 10:1 was selected for assembling the polyplexes (Figure S7, Supporting Information). Subsequently, the size distribution and morphological features of the siRNA‐loaded polyplexes were characterized. As shown in Figure 1b,c, the polyplexes had a size of 54.6 ± 3.5 nm and were observed to be uniform and spherical. Then, the surface of the siRNA‐loaded polyplexes was coated with cholesterol to mask the positive charge, enhancing their stability, and altering the cellular uptake behavior. As shown in Figure 1d, the ζ potential of the nanoparticles gradually decreased with the increasing proportion of added cholesterol. After balancing both the stability and the conjugation ability with drug‐loaded albumin, a 5:1 cholesterol ratio was selected for coating the polyplexes. Finally, the gemcitabine loaded albumin was added, and the exposed N‐hydroxysuccinimides on the polyplexes were used for conjugation, resulting in the final nanoparticle B‐PDEA@ Chole‐Pep‐Alb (CPA). The final nanoparticle had a size distribution of 71.2 ± 6.2 nm, and the TEM image revealed that it consisted of a larger polyplex core and several smaller drug‐loaded albumins (Figure 1e,f). The final nanoparticles B‐PDEA@CPA also showed satisfactory stability in stability assessments (Figure S8, Supporting Information).

To verify that the presence of peptides enables the nanoparticles to respond to overexpressed MMPs in the TME, thereby releasing drug‐loaded albumin and attaining efficient PDAC penetration (Figure 1g), polyplexes and albumin were labeled with FITC and Cy3, respectively. These two fluorophores could trigger fluorescence resonance energy transfer (FRET).^[^ ^46^ ^]^ Thus, the release of drug‐loaded albumin could be evaluated by monitoring the emission spectrum shift under 488 nm excitation. As shown in Figure 1h, with 488 nm excitation, the polyplexes only displayed the emission of FITC, and the albumin exhibited only weak Cy3 emission. However, when conjugated into nanoparticles, a strong Cy3 emission signal was observed, indicating the occurrence of FRET and confirming that the distance between the polyplexes and albumin was within 10 nm, forming intact nanoparticles. After incubation with MMP‐2, the strong FITC emission was restored, while the Cy3 intensity dropped as free albumin, indicating the dissociation of the polyplexes and albumin.

To accomplish efficient gene drug release, 4‐(bromomethyl)‐phenylboronic acid was used to modify the polymer backbone. Triggered by the highly expressed ROS within cancer cells, the carbon‐boron bond would be oxidized, inducing deboronation and releasing p‐quinone methide, which subsequently hydrates to p‐hydroxymethylphenol (HMP) (Figure S10a, Supporting Information). During this process, the polymeric gene carrier undergoes a charge reversal, resulting in the release of the loaded siRNA. This ROS‐responsive deboronation was characterized by ^1^H‐NMR and HPLC (Figures S9 and S10b, Supporting Information). As well as the gene release behavior was confirmed through a gel retardation assay (Figure 1i).

### 2.2 The Cellular Uptake, Selective siRNA Release, and Tumor Penetration of B‐PDEA@CPA

**Figure 2:**
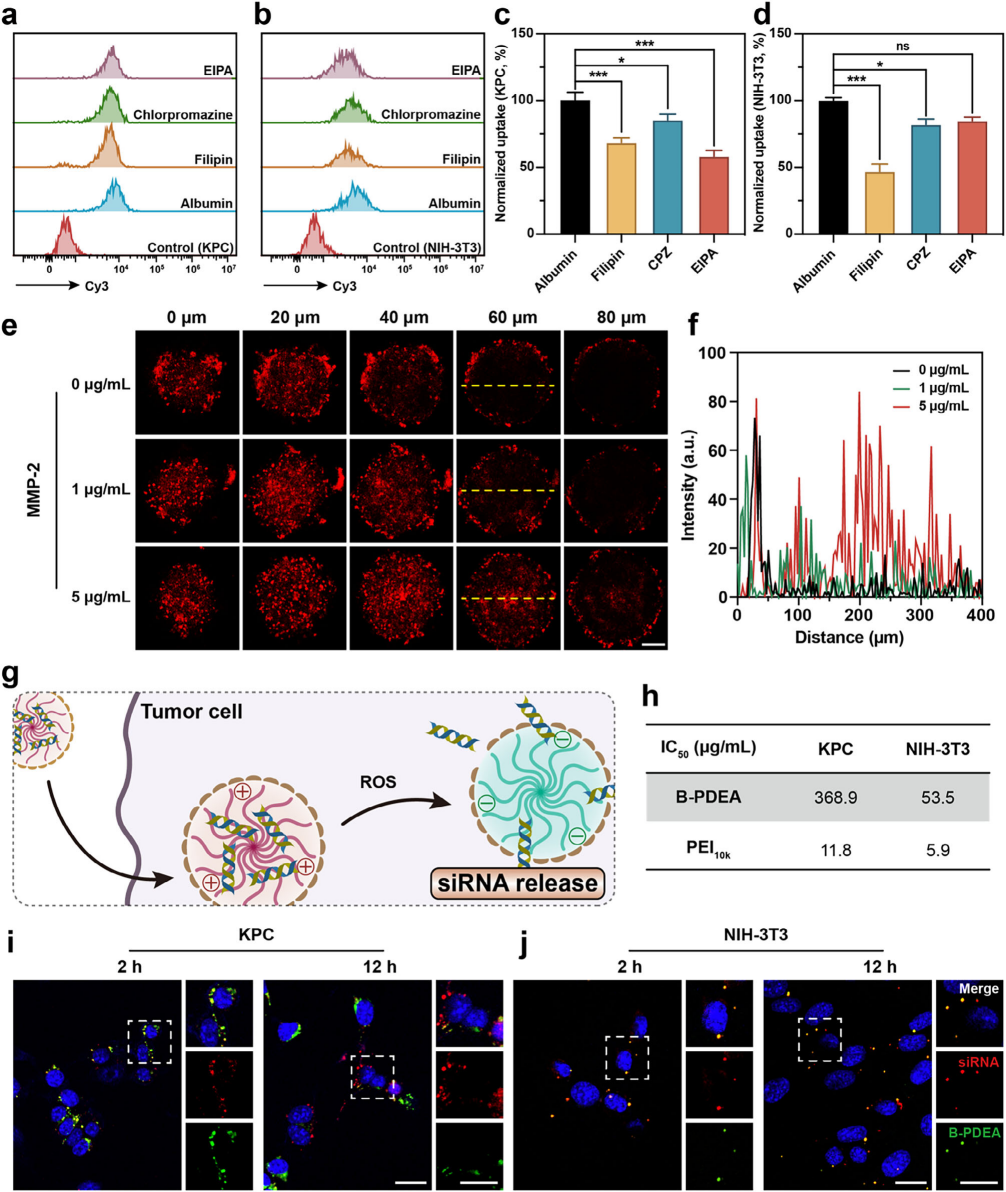
In vitro cellular uptake and siRNA release. FACS analyses of the possible endocytosis pathways of albumin in a) KPC and b) NIH‐3T3 cells by applying different inhibitors. c,d) Semi‐quantification results of a, b). e) CLSM images showing the penetration of MMP‐2 treated nanoparticles in KPC/NIH‐3T3 cocultured MCSs in vitro. The surface of the MCSs was defined as 0 µm. Scale bar = 100 µm. f) Semi‐quantification result of the 60 µm layer images (yellow dash lines). g) Schematic illustration of the ROS triggered charge reversal and siRNA release within tumor cells. h) IC_50_ of KPC or NIH‐3T3 cells incubated with B‐PDEA or PEI_10k_ for 48 h. Intracellular siRNA release and distribution in i) KPC and j) NIH‐3T3 cells. Scale bar = 20 µm. The data are represented as the means ± SD (n = 4). * and *** denote p < 0.01 and p < 0.001. Data were analyzed using one‐way analysis of variance (ANOVA). Prism software package (PRISM 7.0; GraphPad Software, 2016) was applied for all statistical analyses.

KRAS mutations are present in more than 90% of PDAC and orchestrates multiple metabolic changes to promote tumor progression. Notably, the mutation significantly enhances macropinocytosis in PDAC cells.^[^ ^18^, ^47^ ^]^ This receptor‐independent, non‐selective endocytic pathway extensively uptakes extracellular macromolecular nutrients, largely fulfilling the substantial material and energy demands of PDAC. Albumin, a crucial substrate for macropinocytosis, has the potential to load the chemotherapeutic drug gemcitabine and facilitate its uptake by PDAC cells.^[^ ^48^ ^]^ Therefore, inhibitors of different uptake pathways were used to treat KPC cells and fibroblasts (NIH‐3T3) to examine the uptake behavior of albumin in both cell types. As shown in **Figure** **2**a–d, KPC cells exhibited a significantly higher albumin uptake compared to fibroblasts. Additionally, after treatment with filipin and EIPA, the albumin uptake significantly decreased, indicating that the uptake primarily occurs through caveolin‐mediated endocytosis and micropinocytosis in KPC cells. Whereas only filipin effectively inhibited albumin uptake in fibroblasts, demonstrating that enhanced macropinocytosis plays a crucial role in promoting albumin uptake in PDAC cells with KRAS mutation.

In addition to facilitating the cellular uptake of chemotherapeutic drugs, the small particle size of albumin also endows the potential for efficient penetration into the deep area of the compact PDAC tissue, meeting the delivery needs in PDAC treatment. Therefore, a multicellular‐spheroids (MCSs) model was applied to evaluate the tumor penetration ability of albumin. To closely mimic the actual structure of PDAC tissue, MCSs were constructed by co‐culturing KPC cells and fibroblasts at a ratio of 1:9.^[^ ^49^ ^]^ After forming a uniform and compact MCSs, they were co‐incubated with B‐PDEA@CPA under different concentrations of MMP‐2. The distribution and penetration of labeled albumin within the MCSs were tracked (Figure 2e). The Z‐axis section images from CLSM showed that, in the absence of MMP‐2, most of the albumin was scattered only on the surface of the MCSs. However, after MMP‐2 incubation, more albumin was observed in the core regions of the MCSs, and the signal intensity was elevated with MMP‐2 concentrations (Figure 2f). This indicates that the cleavage of the peptide by MMP‐2 leads to the release of small‐sized albumin, enhancing penetration into the MCSs and fulfilling the delivery requirements for PDAC treatment.

Non‐viral gene drug carriers are mostly cationic polymers that attain drug loading through electrostatic interactions between the carriers and the gene drug.^[^ ^50^ ^]^ However, these carriers often retain strong positive charges after loading the genes, which hinders the efficient release of the gene drug upon cellular entry and results in cytotoxicity due to the charge.^[^ ^51^ ^]^ In our design, the innovative gene carrier B‐PDEA can respond to the overexpressed ROS within tumor cells to achieve charge reversal, selectively release gene drugs, and avoid undesired cytotoxicity (Figure 2g). To validate the characteristics of this novel gene carrier, the cytotoxicity of B‐PDEA and the commonly used gene carrier PEI_10k_ in KPC cells and fibroblasts was examined. As shown in Figure 2h, the toxicity of B‐PDEA in both cell types was significantly lower than that of PEI_10k_, demonstrating its superior biocompatibility. Additionally, the difference in cytotoxicity of B‐PDEA between KPC cells and fibroblasts was significantly greater than that of PEI_10k_, which confirmed B‐PDEA can achieve charge reversal within tumor cells and selective gene release. To further confirm the selective release of B‐PDEA within tumor cells, B‐PDEA and siRNA were respectively labeled with fluorescent probes. And their intracellular distribution was tracked at 2 and 12 h post‐administration. As shown in Figure 2i and 2 h after administration, B‐PDEA and siRNA were colocalized, demonstrating that siRNA was encapsulated within the carrier. After 12 h of incubation, colocalization was still observed in fibroblasts. However, within KPC cells, the signals representing B‐PDEA and siRNA had separated, which confirmed the release of siRNA within KPC cells (Figure 2j).

### 2.3 Biodistribution, Tumor Targeting, and Penetration Profiles of B‐PDEA@CPA in the Orthotopic PDAC Model

**Figure 3:**
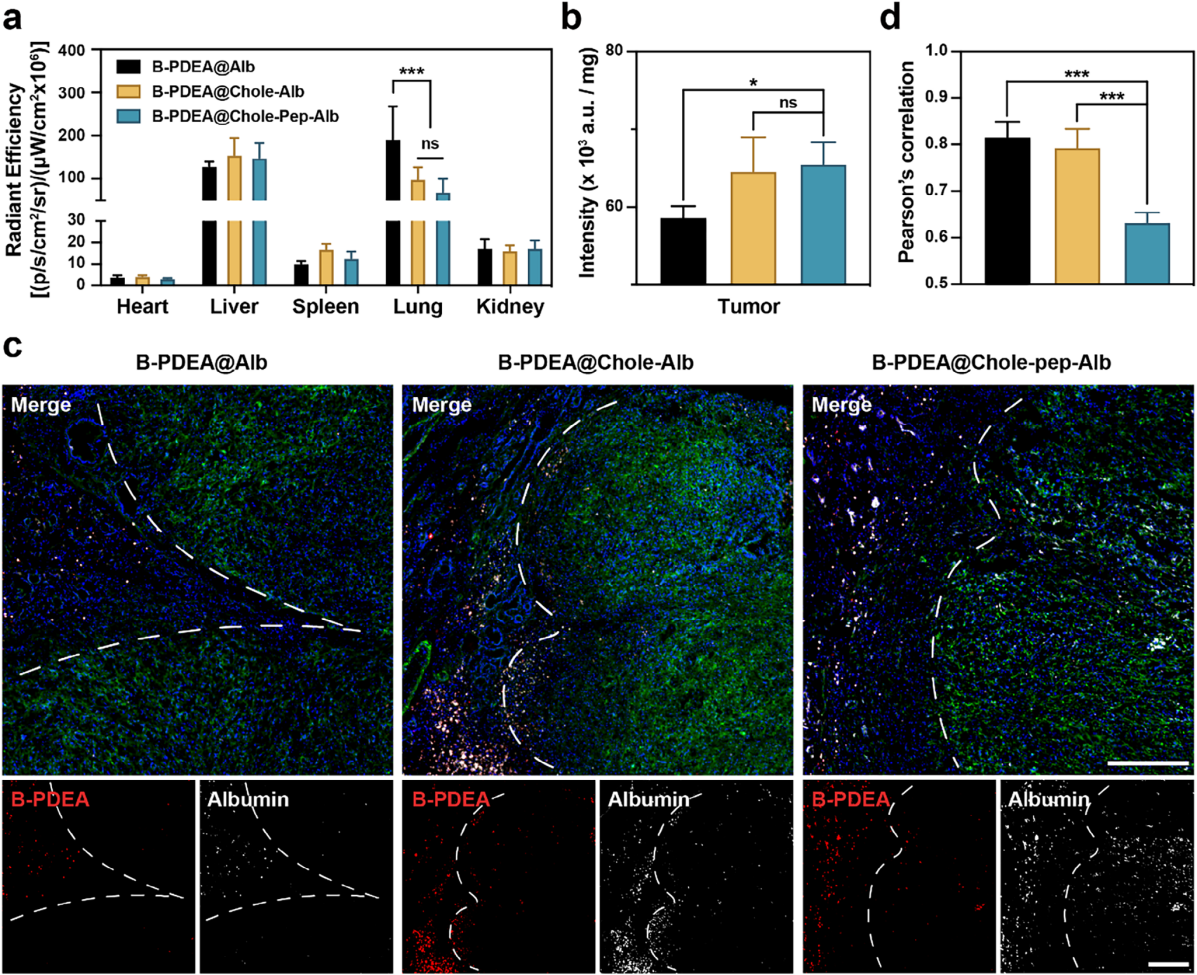
In vivo tumor targeting, distribution, and penetration profiles. a) Biodistribution of labeled nanoparticles in major organs excised from PDAC‐bearing C57 mice. b) Fluorescence intensity of tumor tissues homogenate. c) Representative images of frozen tumor sections from PDAC‐bearing C57 mice. Blue: DAPI for staining cell nucleus; green: Alexa Fluor 488‐labeled Collagen I for staining tumor stroma; red: Cy5.5‐labeled B‐PDEA; white: Cy3‐labeled albumin. Scale bar = 200 µm. d) Pearson's correlation (R) to evaluate the overlap of B‐PDEA and albumin. The data are represented as means ± SD (n = 4). * and *** denote *p* < 0.01 and *p* < 0.001. Data were analyzed using a) two‐way and b, d) one‐way analysis of variance (ANOVA). Prism software package (PRISM 7.0; GraphPad Software, 2016) was applied for all statistical analyses.

An orthotopic PDAC model was constructed to further investigate the targeting and penetration profile of the nanoparticles. The nanoparticles without cholesterol coating (B‐PDEA@Alb), nanoparticles without the responsive peptide (B‐PDEA@Chole‐Alb), and the nanoparticles composed by final formulation (B‐PDEA@Chole‐Pep‐Alb) were injected into tumor‐bearing mice via the tail vein. B‐PDEA polyplexes and albumin were labeled with different fluorescent probes to enable in vivo and *ex vivo* tracking. 24 h post‐injection, the mice were euthanized, and major organs (heart, liver, spleen, lung, kidney) and tumor tissues were collected to track the biodistribution of the nanoparticles (Figures S11 and S12, Supporting Information). As shown in **Figure** **3**a and B‐PDEA@Alb nanoparticles accumulated less in tumor tissues compared to the other two nanoparticles, while significant accumulation was observed in the lungs. This lung accumulation might be caused by the positive charge. Whereas the cholesterol‐coating strategy masked the surface charge and led to optimized biodistribution. This enhanced tumor targeting profile was also confirmed through tumor tissue homogenate (Figure 3b).

Subsequently, to further investigate the specific distribution of nanoparticles within PDAC tissue, the *ex vivo* tumor samples were prepared into frozen sections. The tumor stroma was visualized through immunofluorescence staining for α‐SMA. As shown in Figure 3c and B‐PDEA@Alb displayed less distribution in the tumor, which is consistent with the *ex vivo* imaging and the tumor homogenate. Although the cholesterol coated nanoparticles both exhibited promoted accumulation within the PDAC, there are significant differences in intratumor distribution. The nanoparticles without peptide conjugation (B‐PDEA@Chole‐Alb), both polyplexes and albumin were primarily distributed in the loose tissue at the outside of the tumor. In contrast, the peptide‐conjugated nanoparticles (B‐PDEA@Chole‐Pep‐Alb), most of the albumin penetrated deeply into the compact core of the tumor, while the polyplexes largely remained on the outside. The distribution overlapping of the polyplexes and albumin was quantified with the Pearson correlation coefficient (Figure 3d), which revealed the disintegration of the two components in the peptide‐conjugated nanoparticles. This distribution disparity allows the inhibition of CD39 expression within the stroma and comprehensively modulates the tumor microenvironment. In the meantime, albumins could load the gemcitabine to achieve promoted deep tumor penetration and bring enhanced cytotoxicity.

### 2.4 Microenvironment Modulation and Immune Activation Capacity In Vitro

**Figure 4:**
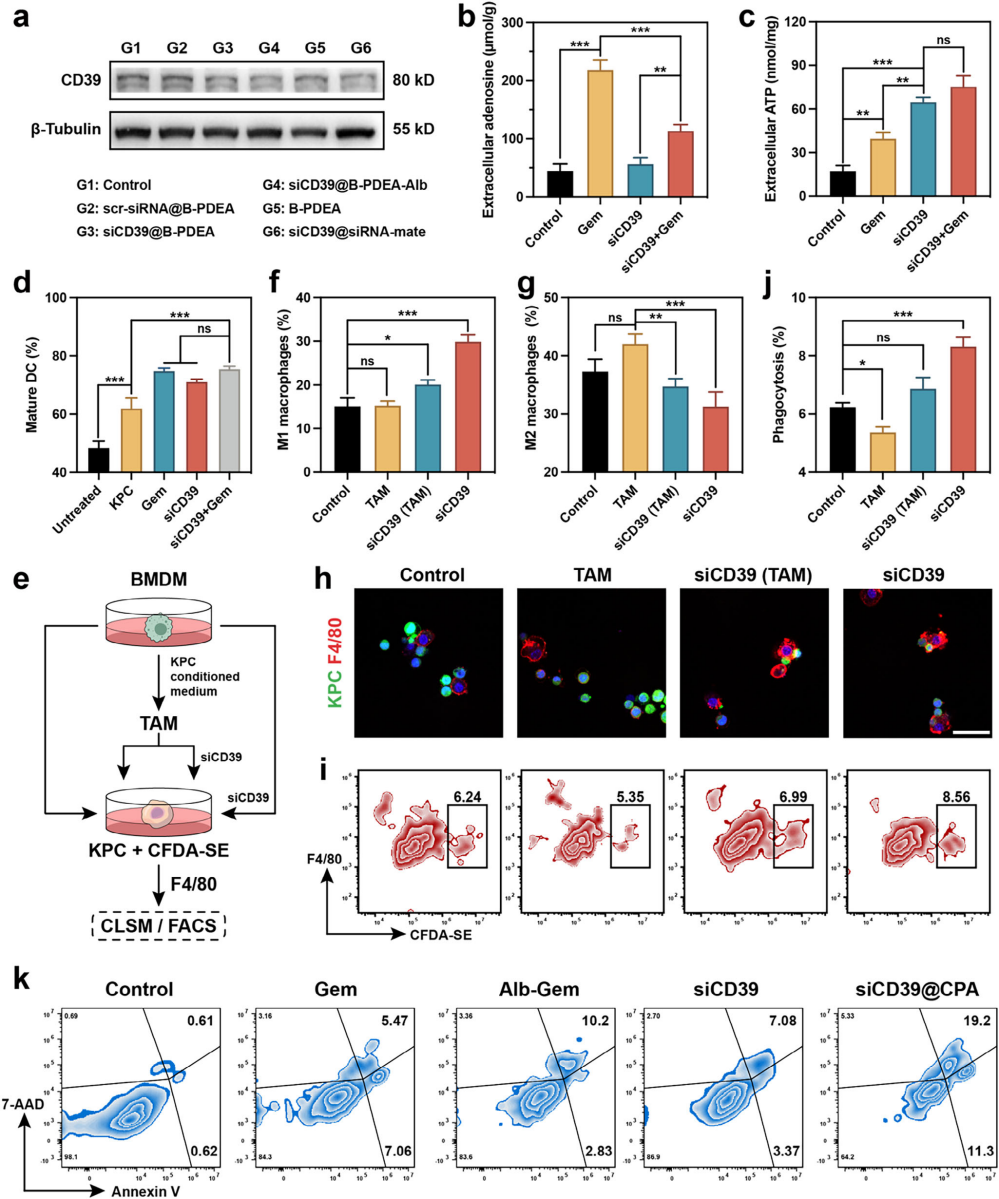
In vitro anti‐tumor and immune activation ability. a) Western blot of CD39 expression in KPC after being treated with different formulations for 48 h. β‐Tubulin was used as the loading control. Extracellular b) adenosine and c) ATP concentration in KPC culture medium after different treatments. d) Statistical result of the FACS analysis of DC maturation after incubating with conditioned media derived from KPC under different treatments. e) Schematic illustration showing the protocol of the BMDM treatment and staining. f and g) Statistical results of the FACS analyses of BMDM polarization after different treatments. h) Representative CLSM images of phagocytosis assay. Scale bar = 40 µm. i) FACS analyses of BMDM phagocytosis against CFDA‐SE labeled KPC cells. j) Semi‐quantification result of i). k) FACS analysis of apoptosis inducing ability with Annexin‐V/7‐AAD staining. The data are represented as means ± SD (n = 4). *, ** and *** denote *p* < 0.01, *p* < 0.005, and *p* < 0.001. Data were analyzed using one‐way analysis of variance (ANOVA). Prism software package (PRISM 7.0; GraphPad Software, 2016) was applied for all statistical analyses.

To evaluate the performance of the gene delivery system in CD39 inhibition, KPC cells were incubated with different nanoparticles for 48 h, and the expression of CD39 was estimated via Western blot. As shown in **Figure** **4**a, there was no noteworthy change in CD39 expression after incubation with drug carrier B‐PDEA or scramble‐siRNA polyplexes. However, after loading CD39 siRNA (siCD39), the expression of CD39 significantly decreased. The CD39 interfering capability of B‐PDEA nanoparticles was consistent with that of the widely used commercial transfection reagent siRNA‐mate, demonstrating the high efficiency of the B‐PDEA delivery system. Notably, the CD39 expression further decreased after incubating with nanoparticles conjugated with albumin, which might be a result of the synergistic effects of chemotherapy and gene therapy. To further confirm that inhibiting CD39 expression can modulate adenosine metabolism, the extracellular adenosine and ATP were quantified with HPLC and a commercial kit. As shown in Figure 4b,c, after incubation with free gemcitabine, the concentrations of extracellular adenosine and ATP both remarkably increased. This might be attributed to the cytotoxicity of gemcitabine, which caused the cell necrosis and the release of ATP. As well as part of the released ATP was converted to adenosine through CD39. It is worth noting that, in the cell culture supernatant treated with both gemcitabine and siCD39, the concentration of adenosine was reduced while the ATP was significantly elevated. This indicates that inhibiting CD39 expression could prevent ATP from being degraded into adenosine, which is conducive to forming a pro‐inflammatory microenvironment and supports the enhanced therapeutic outcome of PDAC.

In TME, extracellular ATP and adenosine play crucial but opposing roles in immune response modulation.^[^ ^52^, ^53^ ^]^ Extracellular ATP would act as danger signals promoting the activation and maturation of dendritic cells and macrophages. Conversely, the ATP metabolite adenosine would induce immunosuppression through binding to A2A receptors, which inhibit dendritic cell maturation and skew macrophages toward a tumor‐promoting M2 phenotype. Therefore, the bone marrow‐derived dendritic cells (BMDCs) were cultured with conditional media from KPC, and treated with gemcitabine or siCD39. Consistent with the level of extracellular ATP and adenosine, more BMDCs were matured (Figure 4d). Besides, the phenotype and function of macrophages were also evaluated. As illustrated in Figure 4e, bone marrow‐derived macrophages (BMDMs) were cultured with KPC derived conditional media and transformed into TAMs, therefore leading to higher detection of the anti‐inflammatory M2 phenotype (Figure 4g). However, following siCD39 treatment, the population of M1 phenotype was elevated and a decreased number of M2 macrophages was observed (Figure 4f). Furthermore, the phagocytosis competence of the macrophages was also assessed. KPC cells were pre‐stained with the long‐term cell fluorescein tracer, and then co‐incubated with the macrophages. As shown in Figure 4h, compared to BMDMs, TAMs manifested reduced phagocytic capacity toward tumor cells. After the treatment with siCD39, the phagocytosis was recovered. This change was also confirmed through flow cytometry (Figure 4i,j). It is demonstrated that through inhibiting CD39, the immunosuppressive effects of adenosine in the TME can be mitigated, leading to improved activation and function of dendritic cells and macrophages.

Moreover, flow cytometry was performed to investigate the apoptosis‐inducing capability of the nanoparticles. As the cell population shown in Figure 4k, free gemcitabine can only cause limited late apoptosis in KPC cells, while albumin conjugated gemcitabine can double this proportion. And the siCD39@CPA could substantially improve the proportion of the late apoptotic tumor cells to 19.2%.

### 2.5 Enhanced Antitumor Efficacy in the Orthotopic PDAC Model

**Figure 5:**
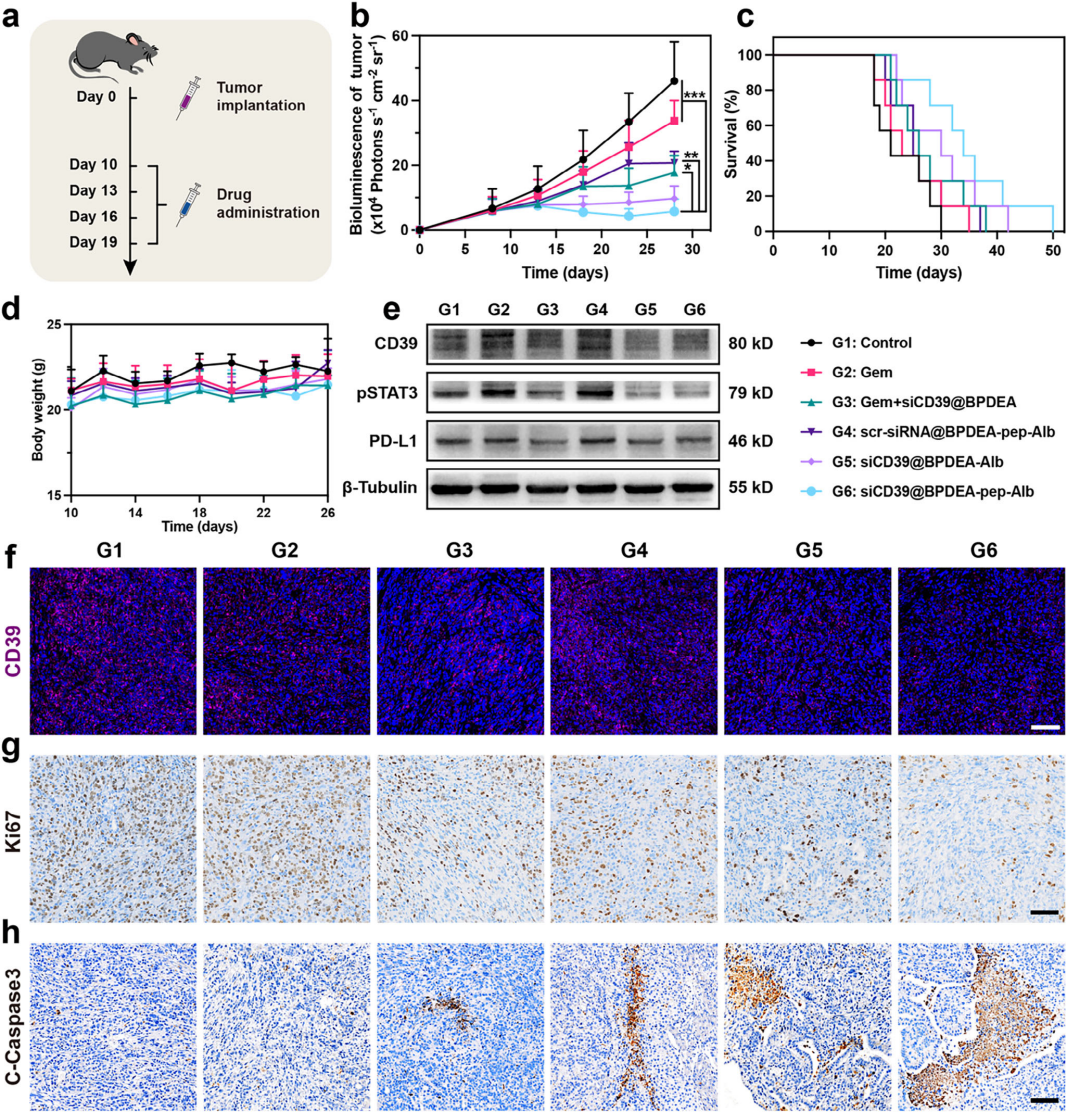
Antitumor efficacy in the orthotopic PDAC model. a) Schematic illustration showing the schedule of drug administration. b) Tumor volume changes measured by the bioluminescence signal (n = 6). c) Survival curves of different groups of treatment (n = 7). d) Body weight recorded 16 days after drug administration (n = 6). e) Western blot of CD39, pSTAT3 and PD‐L1 expression in tumor tissues on day 20^th^. β‐Tubulin was used as the loading control. f) Representative immunofluorescence images of CD39 in different treatment groups. Scale bar = 100 µm. Representative images of immunohistochemical staining of g) Ki67, indicating cell proliferation and h) cleaved‐Caspase 3, indicating cell apoptosis. Scale bar = 100 µm. The data are represented as means ± SD. *, ** and *** denote *p* < 0.01, *p* < 0.005, and *p* < 0.001. Data were analyzed using one‐way analysis of variance (ANOVA). Prism software package (PRISM 7.0; GraphPad Software, 2016) was applied for all statistical analyses.

Encouraged by the remarkable in vitro adenosine metabolism modulating antitumor and immune activating capacity of the nanoparticle, in vivo antitumor and microenvironment modulation efficacy was evaluated with orthotopic PDAC tumor‐bearing male C57BL/6 mice. As illustrated in **Figure** **5**a, 10 days after the tumor implantation, different drugs or nanoparticles were intravenously injected every 3 days for 4 times. The change in body weight was measured and documented every other day (Figure 5d). And since the 8^th^ day after the tumor implantation, the tumor volume of each mouse was monitored by luciferase signal with IVIS every 5 days for 4 times (Figure 5b; Figure S13, Supporting Information). The results showed that among all the treatments, minimal tumor growth as well as the longest survival time was observed in the siCD39@BPDEA‐pep‐Alb treating group (G6), suggesting a promising outcome of the intact nanoparticles (Figure 5b,c). During the treatment, the average body weight of mice in each group was maintained, indicating favorable systematic safety. After completing all four doses of treatment, the treated tumor‐bearing mice from each group were sacrificed, major organs (heart, liver, spleen, lung, and kidney) and tumors were harvested and prepared into paraffin sections. To check if any abnormalities were caused by treatments, Hematoxylin & eosin (H&E) staining was applied (Figures S14 and S15, Supporting Information). The plasma of each group was collected for the test of liver enzyme levels (AST and ALT) and kidney function indicators (BUN and CR) (Figure S16, Supporting Information). Histological structure and biochemical parameters were maintained, implying the favorable biocompatibility of intact nanoparticles.

Antitumor efficacy and mechanism of different treatments were further investigated. As shown in Figure 5e, a significant upregulation of CD39 expression was observed in both Gem (G2) and scr‐siRNA@BPDEA‐pep‐Alb (G4) treating group, emphasizing the contribution of chemotherapy to the deterioration of the aberrant adenosine metabolism, which could be reversed by siCD39 in other treating groups (G3, G5 and G6). Similar result was also observed through immunofluorescence staining (Figure 5f), certifying the CD39 inhibiting capacity of siCD39‐containing nanoparticles. As shown in Figure 5e, the inhibition of STAT3 activation and PD‐L1 expression were also found after treatments,^[^ ^28^ ^]^ suggesting the nanoparticles’ ability of remodeling immune microenvironment and blocking latent pathways of immune escape. In consistency with the tendency of volume growth and survival time extension, the tumor samples of the siCD39@BPDEA‐pep‐Alb treating group (G6) exhibited the lowest proliferation biomarker Ki67 and the strongest apoptosis biomarker Cleaved Caspase3 (C‐Caspase3) signal intensity (Figure 5g,h), showing the optimal antitumor capacity. Also, Sirius red staining and Masson's trichrome staining were employed to visualize the collagen in tumor tissues (Figure S17, Supporting Information). Reductions of collagens were observed, further verifying the stromal modulating effect of metabolic microenvironment modulation.

### 2.6 Immune Responses Promotion in the Orthotopic PDAC Model

KRAS mutations in PDAC result in both the loss of ENTs and the over‐expression of CD39, which synergistically lead to the accumulation of extracellular adenosine in TME. The accumulation of extracellular adenosine contributes greatly to the formation of the immunosuppressive TME, causing DC maturation and macrophage differentiation disorder, as well as T cell function inhibition and the maintenance of regulatory function of regulatory T cells. By the CD39 down‐regulation functioned by siCD39, nanoparticles are expected to reduce extracellular adenosine in TME, meanwhile inducing tumor cells apoptosis and tumor‐derived antigen release through enhanced intracellular gemcitabine delivery, thereby reversing the immunosuppressive TME and reactivating antitumor immunity.

To evaluate the in vivo immune activation capacity of nanoparticles, further investigations were carried out. Tumor tissues and tumor‐draining lymph nodes (TDLNs) of tumor‐bearing mice from each treating group were collected, dispersed into single cells or prepared into sections for FACS or immunofluorescence staining.

**Figure 6:**
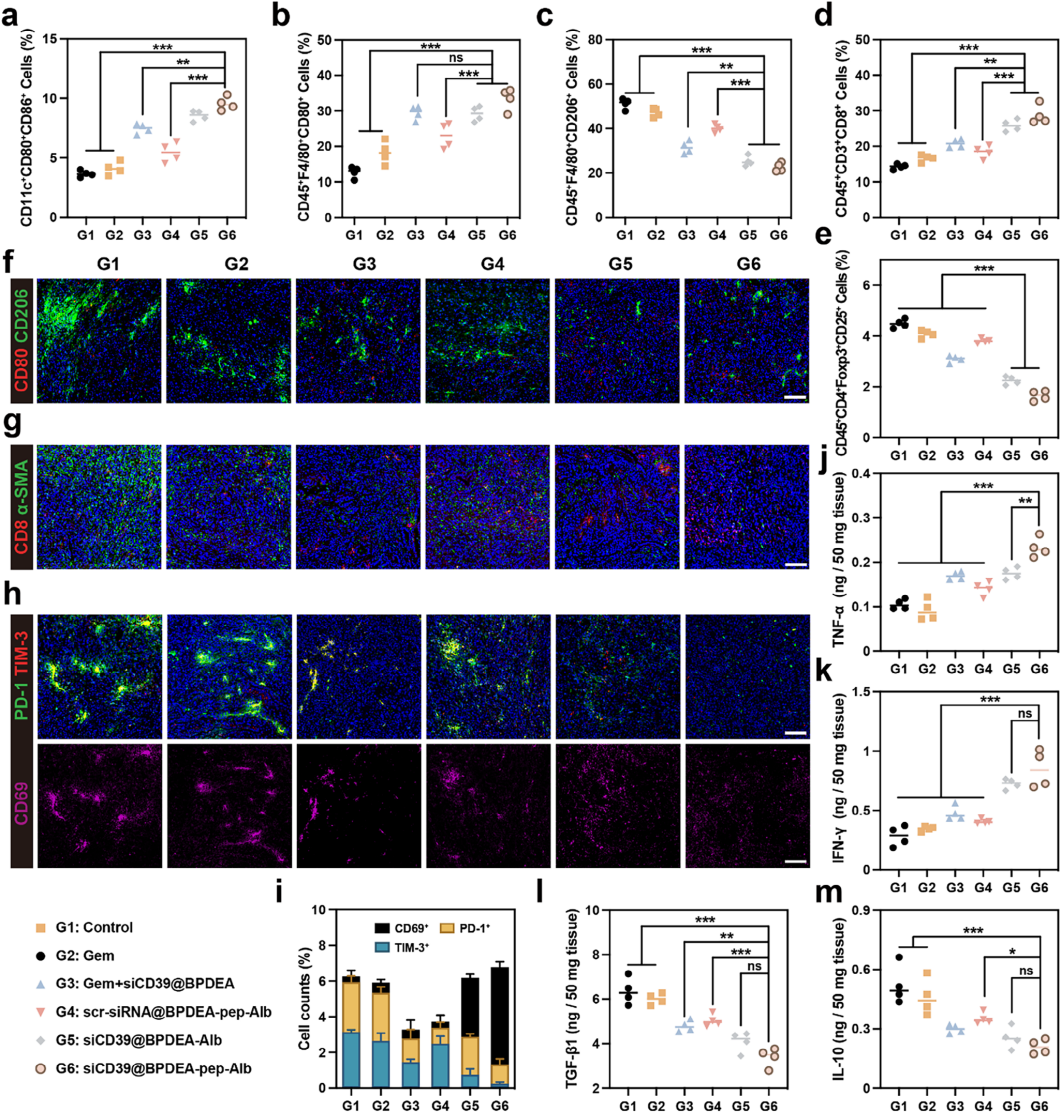
Enhanced antitumor immune responses in the orthotopic PDAC model. a) Statistical result of the FACS analysis of matured DCs (CD11c^+^CD80^+^CD86^+^) in tumor‐draining lymph nodes. b,c) Statistical results of the FACS analyses of M1 macrophages (CD45^+^F4/80^+^CD80^+^), and M2 macrophages (CD45^+^F4/80^+^CD206^+^) infiltrated in orthotopic PDAC tumor tissues. d,e) Statistical results of the FACS analyses of cytotoxic T cells (CD45^+^CD3^+^CD8^+^) and Tregs (CD45^+^CD4^+^Foxp3^+^CD25^+^) infiltrated in orthotopic PDAC tumor tissues. f) Representative immunofluorescence images of M1 macrophages (CD80^+^) and M2 macrophages (CD206^+^) in tumor tissues. Scale bars = 100 µm. g) Representative immunofluorescence images of CD8^+^ T cells infiltration in tumor tissues, green signals indicating tumor stroma labeled with α‐SMA. h) Representative immunofluorescence images of exhausted T cells (PD‐1^+^TIM‐3^+^) infiltration. Scale bars = 100 µm. i) Statistical analysis of the CD69^+^, PD‐1^+^, and/or TIM‐3^+^ cells in orthotopic PDAC tissues. j–m) Expression of TNF‐α, IFN‐γ, TGF‐β1, and IL‐10 in orthotopic PDAC tumors. The data are represented as means ± SD (n = 4). *, **, and *** denote *p* < 0.01, *p* < 0.005, and *p* < 0.001. Data were analyzed using one‐way analysis of variance (ANOVA). Prism software package (PRISM 7.0; GraphPad Software, 2016) was applied for all statistical analyses.

Co‐stimulatory factors CD80 and CD86 are highly expressed on the surface of mature DCs, providing co‐stimulatory signals for T cells and initiate adaptive immune responses through priming T cells.^[^ ^54^ ^]^ Through A2AR mediated cAMP‐PKA axis, adenosine reduces the CD86 expression and cytokine secretion, inhibiting DC maturation and T cell priming.^[^ ^55^ ^]^ In siCD39@BPDEA‐pep‐Alb treating group (G6) we found the highest maturity level of DCs (**Figure** **6**a), confirming the effective activation of immune response initiation. Also, we found that the matured DCs (CD11c^+^CD80^+^CD86^+^) in siCD39@BPDEA‐pep‐Alb treating group (G6) were significantly more than in gemcitabine or scr‐siRNA@BPDEA‐pep‐Alb treating group (G2 or G4), indicating that the promotion of DC maturation by the intact nanoparticles was a multifactorial process, both chemotherapy‐induced antigen release and siCD39‐induced extracellular adenosine reduction might contribute to the maturation of DCs.

By enhancing the production of IL‐10 and arginase in macrophages, adenosine may induce the exacerbation of impaired phagocytic activity and drive M2 polarization without the help of IL‐4Rα.^[^ ^56^, ^57^ ^]^ In consistency with our in vitro results, intact nanoparticle treatment resulted in more pro‐inflammatory M1 macrophages (CD45^+^F4/80^+^CD80^+^) and fewer anti‐inflammatory M2 macrophages (CD45^+^F4/80^+^CD206^+^) infiltration (Figure 6b,c). This conclusion was further confirmed by immunofluorescence staining results shown in Figure 6f.

As a key component of the adaptive immune system, different subtypes of T cells assume different immune functions. We found that drug or nanoparticle treatment led to more cytotoxic T cells (CD45^+^CD3^+^CD8^+^) infiltration in tumor tissues (Figure 6d), which was also confirmed by immunofluorescence staining (Figure 6g). Meanwhile, as one of the main components of tumor ECM, less α‐SMA was observed after treatment, indicating the depletion of intrinsic barriers might be one of the reasons for the increased immune cell infiltration. Also, we found that regulatory T cells (CD45^+^CD4^+^Foxp3^+^CD25^+^, Tregs) notably decreased in the intact nanoparticle treating group (Figure 6e). Considering that adenosine exhibits the ability of promoting the expression of the key transcription factor Foxp3 through the JNK/AP‐1 pathway and maintain its stability by activating A2AR,^[^ ^58^, ^59^ ^]^ the reduction in infiltrating Tregs is believed to be closely related to the changes in adenosine metabolism.

To comprehensively evaluate the activation level of antitumor immunity after treatment, the expression levels of cytokines in tumor tissues were quantified using corresponding ELISA kits. As shown in Figure 6j–m, after treatments, the pro‐inflammatory cytokines TNF‐α^[^ ^60^, ^61^ ^]^ and IFN‐γ^[^ ^62^, ^63^ ^]^ were significantly up‐regulated, while the expression levels of the anti‐inflammatory cytokines TGF‐β^[^ ^64^, ^65^ ^]^ and IL‐10^[^ ^66^ ^]^ were significantly down‐regulated. These evidences further strongly validate the immune activating capabilities of the advanced delivery strategy.

Studies have shown that immune cell exhaustion is a common state of dysfunction of infiltrated immune cells, which is believed to be closely related to tumor development, immune escape and poor immunotherapy outcomes. Considering that T cells are the direct carriers of antitumor immune effects, T cell exhaustion is the most commonly mentioned immune cell exhaustion,^[^ ^67^, ^68^, ^69^ ^]^ which is described as a state characterized by the over‐expression of multiple inhibitory factors, as well as a hierarchical loss of effector functions and memory properties.^[^ ^70^ ^]^ Since adenosine accelerates T cell exhaustion by stimulating the expression of co‐inhibitory factors such as PD‐1, we speculate that by modulating the local aberrant adenosine metabolism, the intact nanoparticle siCD39@BPDEA‐pep‐Alb can improve T cell exhaustion dilemma. To precisely investigate the changes in exhausted T cells, we examined the expression of PD‐1 and TIM‐3 in the CD69^+^ T cells. As expected, in siCD39@BPDEA‐pep‐Alb‐treated tumor tissues, exhausted T cells (CD69^+^PD‐1^+^TIM‐3^+^) were significantly reduced (Figure 6h,i). However, even though the saline and gemcitabine treating groups (G1 and G2) had nearly the same amount of activated T cells, most of them exhibited exhausted phenotype without proper immune functions.

## 3 Conclusion

As the most commonly found gene mutation and main driving force of tumorigenesis, KRAS mutations endow PDAC with extremely strong adaptability and the ability to seek benefits and avoid harm, allowing it to maximize resource and space utilization while excluding external pressure and obtaining chemical and immune privilege. As one of the most typical way, PDAC passively and actively limits gemcitabine uptake and shapes the immunosuppressive TME through aberrant adenosine metabolism, severely inhibiting the activation of the chemotherapy‐immune cascade and leading to its clinical refractory. In this case, our goal is to achieve more chemotherapeutic drug accumulation across barriers and reverse the aberrant metabolic‐immune crosstalk.

Utilizing the endogenous nutrient “access” mechanism, albumin was used as a “Trojan horse” for gemcitabine transport to achieve enhanced chemotherapy drug delivery. Meanwhile, targeting CD39, the key enzyme in the aberrant adenosine metabolic pathway, we designed nanoparticles that co‐deliver gemcitabine and nucleic acid drug siCD39. To match the pathological characteristics of PDAC, the nanoparticles were fabricated with functional modules for tumor targeting, enhanced penetration, and deep‐tissue drug delivery. We demonstrated that the intact siCD39@BPDEA‐pep‐Alb nanoparticles have the expected ability to regulate the aberrant adenosine metabolism, and surprisingly, the intact nanoparticles exhibited the ability to achieve chemotherapy sensitization, metabolism modulation and immunity reactivation, as well as showed excellent antitumor efficacy in the orthotopic PDAC models. This answers the questions found in the clinical regimen. In addition, this delivery system is highly compatible. Through the commercial nanocarrier albumin and the functional positively charged polymer B‐PDEA, our delivery system makes it possible to simultaneously deliver other chemotherapeutic drugs and gene drugs for undruggable targets just like CD39.

## 4 Experimental Section

### Chemicals and Reagents

Gemcitabine and 2‐(N,N‐diethylamino)ethyl methacrylate were ordered from Bide Pharm (Shanghai, China). 6‐maleimidocaproic acid, 2,2′‐azobis(2‐methylpropionitrile) (AIBN) and 4‐(bromomethyl)phenylboronic acid were purchased from Aladdin (Shanghai, China). Hexafluorophosphate azabenzotriazole tetramethyl uranium (HATU), N,N‐diisopropylethylamine (DIPEA) were commercially available from Energy Chemical (Shanghai, China). All anhydrous solvents were purchased from Adamas (Shanghai, China). All other organic solvents were supplied by Sinopharm Chemical Reagent Co., Ltd (Shanghai, China). 4‐cyano‐4‐(phenylcarbonothioylthio)pentanoic acid was ordered from Macklin (Shanghai, China). 4‐cyano‐4‐(phenylcarbonothioylthio)pentanoic acid N‐succinimidyl ester and albumin from human serum (HSA) was commercially available from Sigma‐Aldrich (St. Louis, USA). CD39 siRNA (siCD39, 5′‐GGGCAGAUUCACUCAGGAA‐3′, 5′‐UUCCUGAGUGAAUCUGCCC‐3′) was ordered from Genomeditech (Shanghai, China). Peptide (GPLGVRGC) was purchased from NJPeptide (Nanjing, China)

### Formulation and Characterization of siCD39@CPA Nanoparticles

B‐PDEA, B‐PDEA‐pep‐NHS and siRNA were dissolved in RNase‐free ddH_2_O at various concentrations. B‐PDEA and B‐PDEA‐pep‐NHS solutions were pre‐mixed at the molar ratio of 4:1 to get the working polymer solution. The same volume of siRNA solution was added to the working polymer solution at desired N/P ratio and mixed thoroughly with pipetted for 20 times. The prepared polyplexes were incubated at room temperature for 30 min.

Cholesterol was dissolved in chloroform and added to the eggplant‐shaped flask. The solvent was removed by rotary evaporation to afford a thin lipid film on the flask wall. The film was rehydrated with aforementioned polyplexes solution by ultrasonication for 10 min within ice bath and stirred overnight at room temperature.

HSA (30 mg, 0.45 µmol) dissolved in buffer (PBS 8.0 + 5 mm EDTA), followed by addition of 2‐iminothiolane hydrochloride (Traut's reagent, 0.63 mg, 4.5 µmol) with stirring under nitrogen protection at room temperature for 4 h. The mixture was then centrifuged at 1000 g for 6 min using a desalting column for 3 times, and the product was collected. Gem‐Mal dissolved in DMSO was added to the collected product at desired molar ratio (resulting in a final DMSO concentration of 10%). The mixture was stirred at room temperature overnight, followed by centrifugation at 1000 g for 6 min using a desalting column for 3 times, and the product was collected and freeze‐dried.

The gemcitabine bound HSA was dissolved in ddH_2_O and added to the cholesterol coated polyplexes solution and mixed with pipetted to prepare the final cascade‐targeted nanoparticle siCD39@CPA.

siRNA loading ability was evaluated by agarose gel electrophoresis. The polyplexes were electrophoresed on a 4% agarose gel at 100 V for 30 min. siRNA bands were visualized by GelRed staining. The size and zeta potential of the polyplexes and nanoparticles were measured with Zetasizer Nano ZSP.

### Cell Culture

The murine PDAC cell line KPC (FC1199) was kindly provided by Prof. Jing Xue from Shanghai Cancer Institute. And the murine fibroblast NIH‐3T3 was ordered from Procell (Wuhan, China). The cell lines were cultured with DMEM high glucose medium added 10% fetal bovine serum (for KPC) or 10% newborn calf serum (for NIH‐3T3) and 1% penicillin‐streptomycin under 5% CO_2_ humidified atmosphere at 37 °C. As reported previously, the bone marrow derived dendritic cells and macrophages were isolated through the established protocol.^[^ ^71^ ^]^ Briefly, the femurs and tibias were collected from the euthanized C57BL/6 mice and sterilized with 75% ethanol. The shaft of each femur or tibia was rinsed repeatedly with 3 mL RPMI 1640 medium containing 10% fetal bovine serum, 1% penicillin‐streptomycin, 20 ng mL^−1^ GM‐CSF, and 10 ng mL^−1^ IL‐4 (Sino Biological, Beijing, China). The remained marrow tissues were removed with a 100 µm cell filter. After 3 days of culture, the cells were rinsed gently with a pipette, and the adherent cells were cultured for another 4 days and collected as bone marrow derived macrophages. Also, the supernatant was collected and cultured in a new petri dish for another 4 days to acquire bone marrow derived dendritic cells.

### Animals and Orthotopic PDAC Model Establishment

Male C57BL/6 mice were provided by Shanghai Institute for Biomedical and Pharmaceutical Technologies. All the animals were held and the experiments were performed in conformity with guidelines evaluated and approved by Fudan University Institutional Animal Care and Use Committee (2022‐03‐YJ‐ST‐10).

The orthotopic PDAC animal model was established by injecting 75 µL HBSS containing 1.0 × 10^6^ KPC‐Luci cells into the pancreas of C57BL/6 mice. The growth of the tumor was monitored by IVIS spectrum (Caliper PerkinElmer, Waltham, USA).

### Preparation of KPC/NIH‐3T3 Cocultured MCSs

Preparation of MCSs culture medium: methyl cellulose powder (6 g) treated with high pressure steam was stirred in 250 mL DMEM medium (preheated to 60 °C). Then 250 mL cold DMEM medium containing 20% FBS was added and mixed at room temperature, resulting in a final volume of 500 mL. Then the mixture was stirred overnight at 4 °C. Centrifugation (5000 rpm, RT, 2 h) was applied to obtain supernatant for further application.

Cell suspension (10^6^ cells mL^−1^, KPC/NIH‐3T3 = 1/9) in DMEM medium containing 10% FBS was added with 20% spheroids culture medium to obtain MCSs stock solution. The stock solution was placed on the lid of cell culture dish drop by drop (20 µL drop^−1^). HBSS was then poured into the dish. The dish was covered with the lid, and cultured for 3 days (until the medium turns yellow), then visible KPC/NIH‐3T3 MCSs could be obtained.

### Confocal Microscopy of KPC/NIH‐3T3 Cocultured MCSs

The albumin in the nanoparticle was pre‐labeled with Cyanine3. And the aforementioned MCSs were treated with different labeled nanoparticles under various MMP‐2 concentrations for 6 h. Then, the MCSs were washed with HBSS for three times, fixed with 4% formaldehyde for 30 min and further stained with DAPI for 15 min. The treated MCSs were subjected to confocal fluorescence microscope (IX2‐RFACA, Olympus, Osaka, Japan) for 3D analysis.

### Cytotoxicity and Cellular Apoptosis Inducing Assay

The cell viability was measured with the cell counting kit 8 (CCK‐8, Meilun Biotech, Dalian, China). 2.0 × 10^3^ KPC cells or 5.0 × 10^3^ NIH‐3T3 cells were seeded into each well of 96‐well plates, respectively, and corresponding concentrations of B‐PDEA or PEI_10k_ was added into each well and incubated at 37 °C for 48 h. The culture media were replaced with HBSS containing 10% CCK‐8 and incubated for another 1 h at 37 °C. The absorbance was measured by a microplate reader (Thermo Fisher Scientific, Eugene, USA) at 450 nm.

To evaluate the cellular apoptosis inducing ability, 1.0 × 10^5^ KPC cells per well were seeded into 6‐well plates and cultured overnight. Free drugs or nanoparticles were added into corresponding wells and incubated at 37 °C for 24 h. After the treatment, the cells were removed from the plates with EDTA‐free trypsin and stained with an apoptosis detection kit (Annexin V‐PE, 7‐AAD) (Meilun Biotech, Dalian, China) as instructed. Then the stained cells were analyzed with the flow cytometer.

### Western Blot

The proteins from cultured cells or tissues were extracted with Western cell lysis buffer or RIPA lysis buffer (Beyotime Biotechnology, Shanghai, China), respectively. The protease and phosphatase inhibitors were added as required. The total protein concentration was measured with a BCA kit (Beyotime Biotechnology, Shanghai, China) and diluted to the same concentration for further analysis. The proteins were separated with SDS‐PAGE gradient gel (Beyotime Biotechnology, Shanghai, China) and transferred to the 0.45 µm PVDF membrane (Merck, Rahway, USA). The specific proteins were detected by corresponding antibodies. Anti‐CD39 and anti‐PD‐L1 antibodies were ordered from ABclonal Technology (Wuhan, China). Anti‐pSTAT3 antibody was ordered from Cell Signaling Technology (Boston, MA, United States). And the HRP‐conjugated anti‐β‐Tubulin antibodies were purchased from Yeasen Biotechnology (Shanghai, China).

### In Vivo Antitumor Capacity Evaluation

Ten days after the tumor implantation, the mice were divided into six groups randomly (n = 10). And the mice received the treatment of saline, free gemcitabine and siCD39@BPDEA (in which gemcitabine was not chemically bound to the nanoparticle), scr‐siRNA@BPDEA‐pep‐Alb (in which siRNA was scrambled that could not exhibit CD39 inhibiting capacity), siCD39@BPDEA‐Alb (nanoparticle without MMP‐2 cleavable peptide) and siCD39@BPDEA‐pep‐Alb at the equal dose of 5 mg kg^−1^ calculated by gemcitabine and 0.25 mg kg^−1^ calculated by siRNA through intravenous injection. The tumor growth was monitored with IVIS spectrum every 5 days for 4 times, and the body weight of each mouse was recorded every other day for 20 days. On Day 20^th^, the major organs and tumor tissues were harvested and prepared as paraffin sections for further investigation. The blood samples were also collected to evaluate the biocompatibility of the nanoparticles.

### Flow Cytometry of Ex Vivo Samples

To explore the infiltration of immune cells and the maturation of DCs, the tumor tissues and lymph nodes of the orthotopic PDAC‐bearing mice were harvested. The *ex vivo* tissues were cut into small pieces and squeezed through 70 µm cell filters to prepare single cell suspension, then diluted to 1.0 × 10^6^ cells mL^−1^ in HBSS for further staining. The detailed staining strategy was the same as reported before.^[^ ^42^, ^72^ ^]^ The gating strategies were presented in Figures S14–S17, (Supporting Information). All fluorescence probe‐conjugated antibodies used in *ex vivo* flow cytometry were commercially available from Thermo Fisher Scientific (Eugene, USA). All the antibodies were presented as following: Cytotoxic T cells (anti‐CD45‐APC, eBioscience, Catalog 17‐0451‐82; anti‐CD3‐FITC, eBioscience, Catalog 11‐0032‐82; anti‐CD8a‐PE, eBioscience, Catalog 12‐0081‐82); Tregs (anti‐CD45‐APC, eBioscience, Catalog 17‐0451‐82; anti‐CD4‐PerCP‐Cyanine5.5, eBioscience, Catalog 45‐0042‐82; anti‐CD25‐ Alexa Fluor 488, eBioscience, Catalog 53‐0251‐82; anti‐Foxp3‐eFlour 450, eBioscience, Catalog 48‐5773‐80); Macrophages (anti‐CD45‐APC, eBioscience, Catalog 17‐0451‐82; anti‐F4/80‐FITC, eBioscience, Catalog 11‐4801‐82; anti‐CD80‐PE‐Cyanine5.5, eBioscience, Catalog 15‐0801‐81; anti‐CD206‐Alexa Flour 700, eBioscience, Catalog 56‐2061‐82); DCs (anti‐CD11c‐PerCP‐Cyanine5.5, eBioscience, Catalog 45‐0114‐82; anti‐CD80‐APC, eBioscience, Catalog 17‐0801‐82; anti‐CD86‐PE, eBioscience, Catalog 12‐0862‐82).

### Statistical Analysis

Prism software package (PRISM 7.0; GraphPad Software, 2016) was applied for all statistical analyses. All data were analyzed using one‐way analysis of variance (ANOVA) or two‐way analysis of variance (ANOVA). The data were represented as means ± SD. The sample sizes (n) in different experiments were mentioned in figure legends. *p* value < 0.05 was considered statistically significant. *, **, and *** denoted *p* < 0.01, *p* < 0.005, and *p* < 0.001.

## Conflict of Interest

The authors declare no conflict of interest.

## Author Contributions

H.F. and H.C. contributed equally to this work. H.F. and H.C. performed experiments, analyzed data and wrote the manuscript. C.J. supervised and provided funding for this project. Other authors have provided valuable suggestions for this project.

## Supporting information



Supporting Information

## Data Availability

Research data are not shared.
