# Supplementary material for: Cascade‐Targeted Nanoparticles for Enhanced Gemcitabine Delivery and Adenosine Metabolism Modulation to Overcome Treatment Resistance in Pancreatic Cancer
Source: Adv Sci (Weinh). 2025 Jul 8;12(38):e07118. doi: 10.1002/advs.202507118 (PMC12520539; doi:10.1002/advs.202507118)
Supplement: Supplementary file 1 — Supporting Information [file ADVS-12-e07118-s001.docx]

Supporting Information

**Cascade-Targeted Nanoparticles for Enhanced Gemcitabine Delivery and Adenosine Metabolism Modulation to Overcome Treatment Resistance in Pancreatic Cancer**

*Hongrui Fan^1#^, Hongyi Chen^1#^, Haolin Song^1^, Chufeng Li^1^, Yu Wang^1^, Zhenhao Zhao^1^, Qin Guo^1^, Xuwen Li^1^, Mingxuan Liu^1^, Tao Sun^1*^ and Chen Jiang^1^****^*^***

^1^ Department of Pharmaceutics, School of Pharmaceutical Sciences, Fudan University; Key Laboratory of Smart Drug Delivery, Ministry of Education; State Key Laboratory of Brain Function and Disorders and MOE Frontiers Center for Brain Science, Shanghai 201203, China.

*^#^* These authors contributed equally to this work.

*^*^* Corresponding author

E-mail: jiangchen@shmu.edu.cn (Chen Jiang); sunt@fudan.edu.cn (Tao Sun)

**Contents:**

Synthesis of Gem-Mal, B-PDEA and B-PDEA-pep-NHS

**Scheme S1.** Synthesis route of **Gem-Mal**.

**Scheme S2.** Synthesis route of **B-PDEA**.

**Scheme S3.** Synthesis route of **B-PDEA-pep-NHS**.

**Figure S1.** The ^1^H NMR spectrum of **Gem-Mal** in DMSO-d6.

**Figure S2.** The ^1^H NMR spectrum of **PDEA** in CD_3_OD.

**Figure S3.** The ^1^H NMR spectrum of **B-PDEA** in CD_3_OD.

**Figure S4.** The ^1^H NMR spectrum of **RAFT-pep-NHS** in DMSO-d6.

**Figure S5.** The ^1^H NMR spectrum of **PDEA-pep-NHS** in CD_3_OD.

**Figure S6.** The ^1^H NMR spectrum of **B-PDEA-pep-NHS** in CD_3_OD.

**Figure S7.** Agarose gel electrophoresis of polyplexes at different N/P ratios.

**Figure S8.** Size and PDI changes of B-PDEA@CPA nanoparticles upon incubating with a) PBS (pH 7.4) and b) PBS contain 10% FBS for a week. The data are represented as the mean ± SD (n = 3).

**Figure S9.** The HMP release traced by HPLC of **B-PDEA** incubated with 1 mM H_2_O_2_ at 37 °C.

**Figure S10.** The ROS-responsive profile of **B-PDEA**. a) Scheme of the ROS-triggered charge reversal of **B-PDEA**. b) ^1^H-NMR spectra of **B-PDEA** after treated with different concentration of H_2_O_2_ and self-catalyzed hydrolysis in D_2_O.

**Figure S11.** *In vivo* luminescence and fluorescent images of B-PDEA@Alb, B-PDEA@Chole-Alb and B-PDEA@Chole-Pep-Alb post 1, 4, 10, and 24 h i.v. injection.

**Figure S12.** Biodistribution of B-PDEA@Alb, B-PDEA@Chole-Alb and B-PDEA@Chole-Pep-Alb post 24 h i.v. injection in main organs and tumors.

**Figure S13.** Tumor volume changes of each treating group measured by the bioluminescence signal.

**Figure S14.** Representative images of H&E staining of major organs (heart, liver, spleen, lung, and spleen) from KPC xenograft mice treated with different formulations of Day 20^th^. Scale bar = 500 μm.

**Figure S15.** Slide scan images of H&E staining of major organs (heart, liver, spleen, lung, and spleen) from KPC xenograft mice treated with different formulations of Day 20^th^. Scale bar = 2 mm.

**Figure S16.** Liver enzyme levels (AST and ALT) and kidney function indicators (CR and BUN) in mice treated with different formulations of Day 20^th^. The data are represented as the mean ± SD (n = 3). The green shading indicates the normal ranges of each indicator. AST, aspartate aminotransferase; ALT, alanine aminotransferase; BUN, blood urea nitrogen; CR, serum creatinine.

**Figure S17.** a) Representative polarized images of Sirius Red staining indicating collagen in tumor tissues. Scale bar = 500 μm. b) Representative images of Masson’s trichrome staining indicating collagen in tumor tissues. Scale bar = 200 μm.

**Figure S18.** Gating strategy to determine frequencies of CD8^+^ T cells. Representative FACS analysis plots of cytotoxic T cells (CD45^+^CD8^+^) infiltrated in orthotopic PDAC tumors.

**Figure S19.** Gating strategy to determine frequencies of regulatory T cells (Tregs). Representative FACS analysis plots of Tregs (CD45^+^CD4^+^CD25^+^Foxp3^+^) infiltrated in orthotopic PDAC tumors.

**Figure S20.** Gating strategy to determine frequencies of M1/M2 macrophages. Representative FACS analysis plots of M1 macrophages (CD45^+^F4/80^+^CD80^+^) and M2 macrophages (CD45^+^F4/80^+^CD206^+^) infiltrated in orthotopic PDAC tumors.

**Figure S21.** Gating strategy to determine frequencies of mature DCs. Representative FACS analysis plots of mature DCs (CD11c^+^CD80^+^CD86^+^) in tumor-draining lymph nodes.

**Synthesis of Gem-Mal, B-PDEA and B-PDEA-pep-NHS**

The gemcitabine prodrug, ROS-responsive polymers and polymer-peptide conjugate were synthesized following the designed procedure.


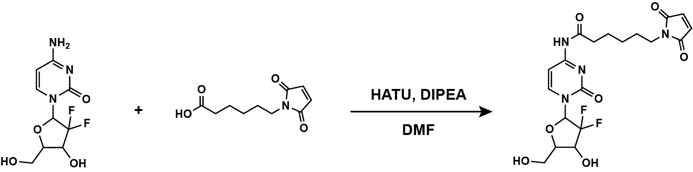


**Scheme S1.** Synthesis route of **Gem-Mal**.

Gemcitabine (100 mg, 0.38 mmol), 6-maleimidocaproic acid (160.5 mg, 0.76 mmol) hexafluorophosphate azabenzotriazole tetramethyl uranium (HATU, 361.2 mg, 0.95 mmol), N,N-diisopropylethylamine (DIPEA, 166 μL, 0.95 mmol) was dissolved in dry DMF (10 mL). The mixture was stirred for 48 h under room temperature. The reaction was monitored by TLC (MeOH:DCM = 1:10). After removal of solvent, the residual was purified by column chromatography on silica gel (MeOH:DCM = 1:10) to give **Gem-Mal** in the form of white solid.

^1^H NMR (400 MHz, DMSO-d6) δ 8.24 (d, J = 7.6 Hz, 1H), 7.27 (d, J = 7.7 Hz, 1H), 7.00 (d, J = 1.6 Hz, 2H), 6.34 (d, J = 6.5 Hz, 1H), 6.17 (t, J = 7.5 Hz, 1H), 5.33 (d, J = 5.6 Hz, 1H), 4.25 - 4.11 (m, 1H), 3.88 (dt, J = 8.8, 2.9 Hz, 1H), 3.80 (d, J = 12.9 Hz, 1H), 3.68 - 3.61 (m, 1H), 2.38 (t, J = 7.4 Hz, 2H), 1.51 (dp, J = 22.0, 7.4 Hz, 4H), 1.19 (dd, J = 13.0, 6.3 Hz, 2H).


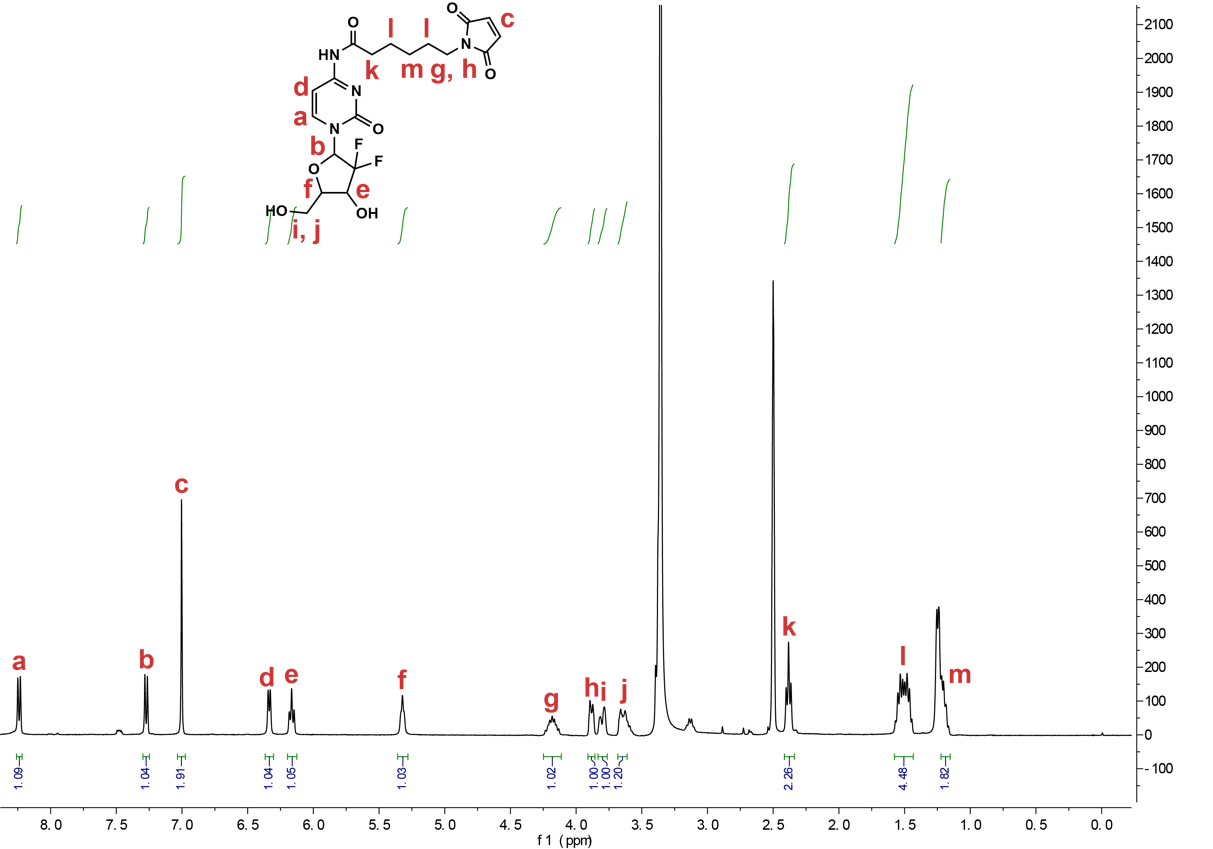


**Figure S1.** The ^1^H NMR spectrum of **Gem-Mal** in DMSO-d6.


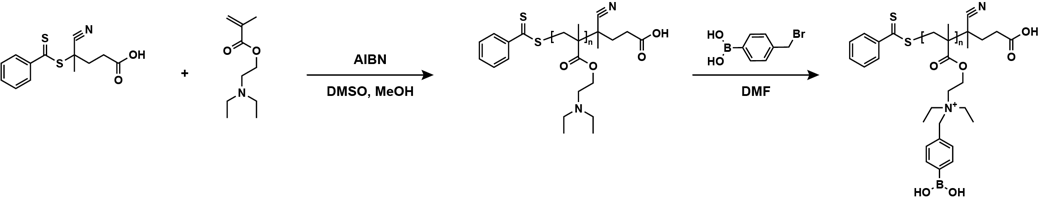


**Scheme S2.** Synthesis route of **B-PDEA**.


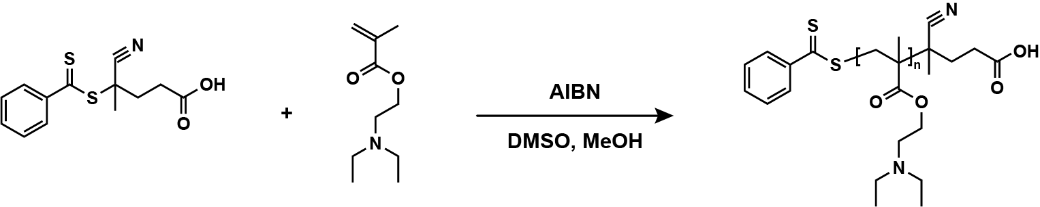


4-cyano-4-(phenylcarbonothioylthio)pentanoic acid (10.6 mg, 0.038 mmol), 2-(N,N-diethylamino)ethyl methacrylate (645.2 mg, 3.48 mmol), and 2,2'-azobis(2-methylpropionitrile) (AIBN, 4.92 mg, 0.03 mmol) were dissolved in dry MeOH (2.5 mL), and added in a Schlenk tube. The solution was degassed through five repeated freeze-pump-thaw cycles with liquid nitrogen. The polymerization was carried out at 70 °C for 16 h, after completion the reaction was quenched by opening the tube, concentrated under reduced pressure, and precipitated with cold n-hexane for 3 times to give **PDEA** in the form of salmon thick liquid.

^1^H NMR (400 MHz, CD_3_OD) δ 4.06 (s, 164H), 2.79 (q, J = 7.8 Hz, 157H), 2.64 (q, J = 7.5 Hz, 1 321H), 1.91 (d, J = 30.8 Hz, 144H), 1.10 (t, J = 7.1 Hz, 564H), 0.93 (d, J = 10.0 Hz, 149H).


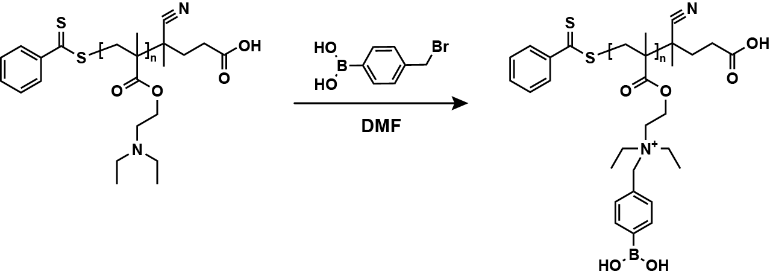


PDEA (85 mg, 5.7 μmol), 4-(bromomethyl)phenylboronic acid (165.3 mg, 0.77 mmol), and anhydrous K_2_CO_3_ (110.6 mg, 0.80 mmol) were dissolved in dry DMF (5 mL), and stirred for 24 h under an argon atmosphere at room temperature. The mixture was dialyzed for 24 h against deionized water (3.5 k MWCO). Lyophilization was applied for dehydration to give **B-PDEA** in the form of flocculent off-white solid.

^1^H NMR (400 MHz, CD_3_OD) δ 7.72 (s, 168H), 7.38 (s, 164H), 4.54 (d, J = 13.1 Hz, 160H), 3.60 (s, 484H), 1.93 (s, 142H), 1.56 - 0.86 (m, 729H).


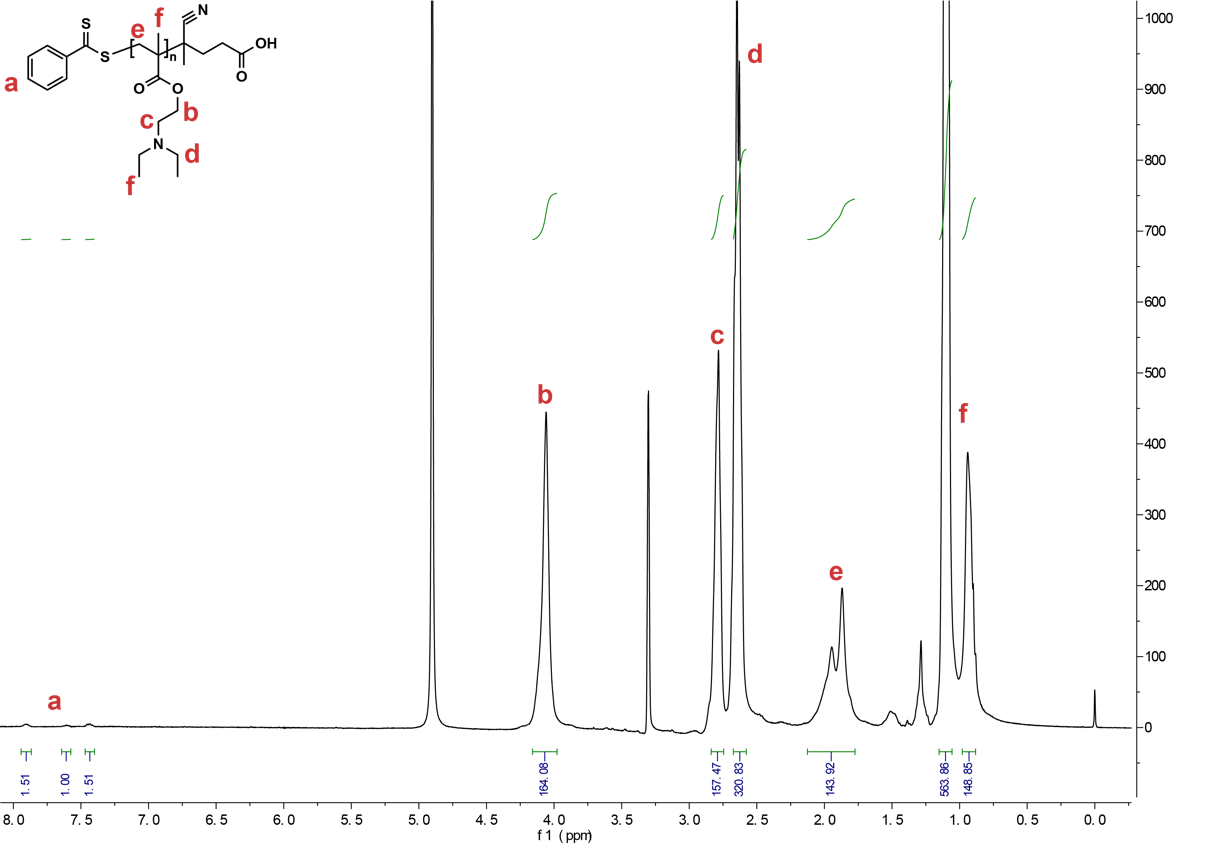


**Figure S2.** The ^1^H NMR spectrum of **PDEA** in CD_3_OD.


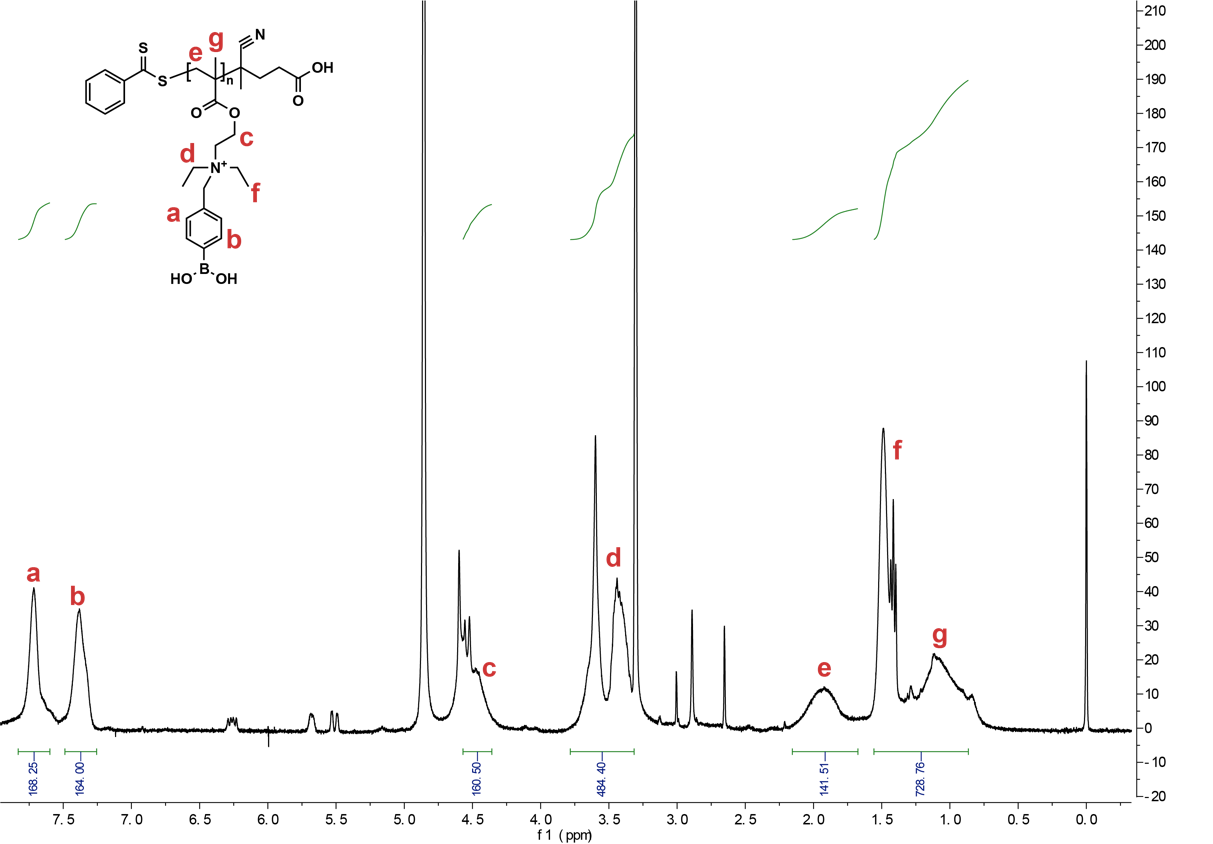


**Figure S3.** The ^1^H NMR spectrum of **B-PDEA** in CD_3_OD.


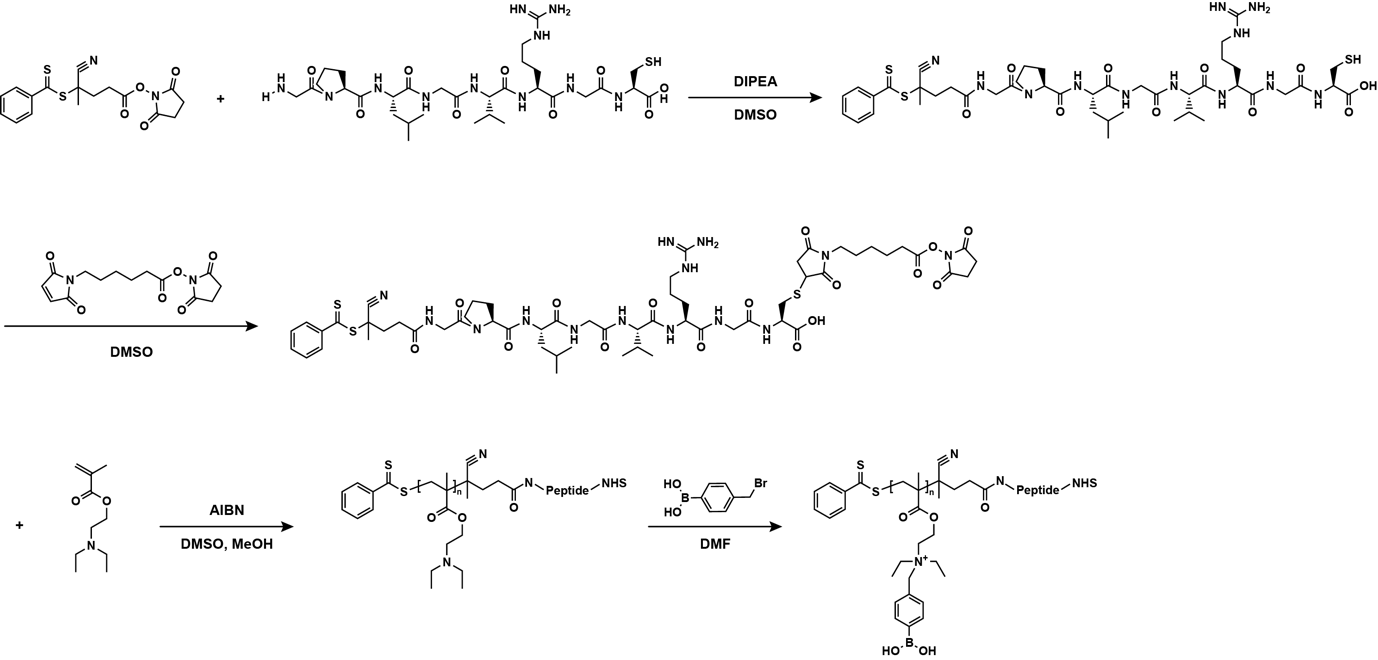


**Scheme S3.** Synthesis route of **B-PDEA-pep-NHS**.


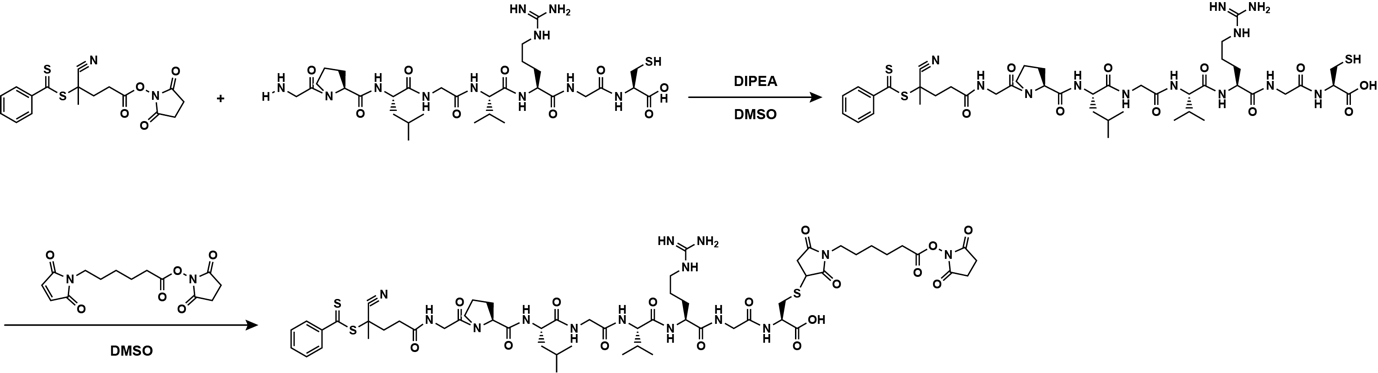


Peptide (GPLGVRGC, 8 mg, 10.6 μmol) and DIPEA (3 μL, 17.2 μmol) were dissolved in dry DMF (1 mL). 4-cyano-4-(phenylcarbonothioylthio)pentanoic acid N-succinimidyl ester (5.3 mg, 14.1 μmol) in dry DMSO (1 mL) was added dropwise in 15 min, the mixture was stirred for another 4 h under room temperature. N-Succinimidyl 6-maleimidohexanoate (4.6 mg, 14.9 μmol) in dry DMSO (1 mL) was added dropwise in 15 min, and stirred for another 4 h under room temperature. The mixture was dialyzed for 24 h against DMSO (1 k MWCO) for 24 h and deionized water (1 k MWCO) for another 24 h. Lyophilization was applied for dehydration to give **RAFT-pep-NHS** in the form of flocculent dark pink solid.

^1^H NMR (400 MHz, DMSO-d6) δ 7.90 (dd, J = 11.9, 7.8 Hz, 2H), 7.49 (dt, J = 15.2, 7.7 Hz, 3H), 4.26 (d, J = 36.8 Hz, 4H), 3.92 - 3.64 (m, 4H), 2.54 (s, 4H), 1.53 (d, J = 35.9 Hz, 6H), 0.81 (td, J = 17.4, 6.3 Hz, 12H).


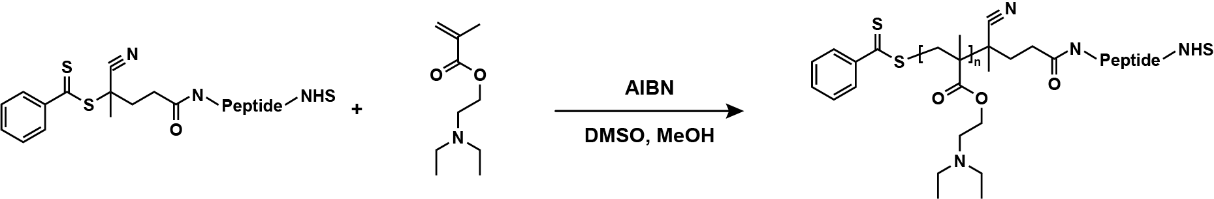


RAFT-pep-NHS (26.3 mg, 19.8 μmol), 2-(N,N-diethylamino)ethyl methacrylate (366.8 mg, 1.98 mmol), and 2,2'-azobis(2-methylpropionitrile) (AIBN, 3.7 mg, 0.02 mmol) were dissolved in dry MeOH (2 mL), and added in a Schlenk tube. The solution was degassed through five repeated freeze-pump-thaw cycles with liquid nitrogen. The polymerization was carried out at 70 °C for 16 h, after completion the reaction was quenched by opening the tube, concentrated under reduced pressure, and precipitated with cold n-hexane for 3 times to give **PDEA-pep-NHS** in the form of sticky pale pink liquid.

﻿^1^H NMR (400 MHz, CD_3_OD) δ 4.13 (s, 125H), 2.86 (d, J = 6.6 Hz, 123H), 2.71 (q, J = 8.0 Hz, 277H), 1.98 (d, J = 30.6 Hz, 112H), 1.18 (q, J = 7.3 Hz, 439H), 1.00 (s, 128H).


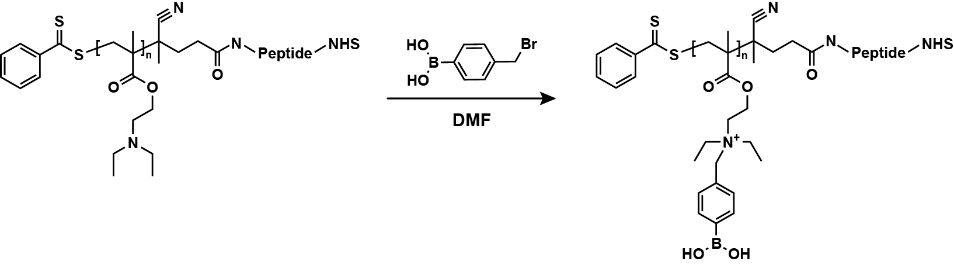


PDEA-pep-NHS (50 mg, 3.2 μmol), 4-(bromomethyl)phenylboronic acid (77.3 mg, 0.36 mmol), and anhydrous K_2_CO_3_ (55.3 mg, 0.40 mmol) were dissolved in dry DMF (3 mL), and stirred for 24 h under an argon atmosphere at room temperature. The mixture was dialyzed for 24 h against deionized water (3.5 k MWCO). Lyophilization was applied for dehydration to give **B-PDEA-pep-NHS** in the form of flocculent off-white solid.

^1^H NMR (400 MHz, CD_3_OD) ﻿δ 7.71 (s, 127H), 7.37 (s, 124H), 4.53 (d, J = 13.3 Hz, 117H), 3.52 (d, J = 68.0 Hz, 364H), 1.94 (s, 108H), 1.61 - 0.79 (m, 551H).


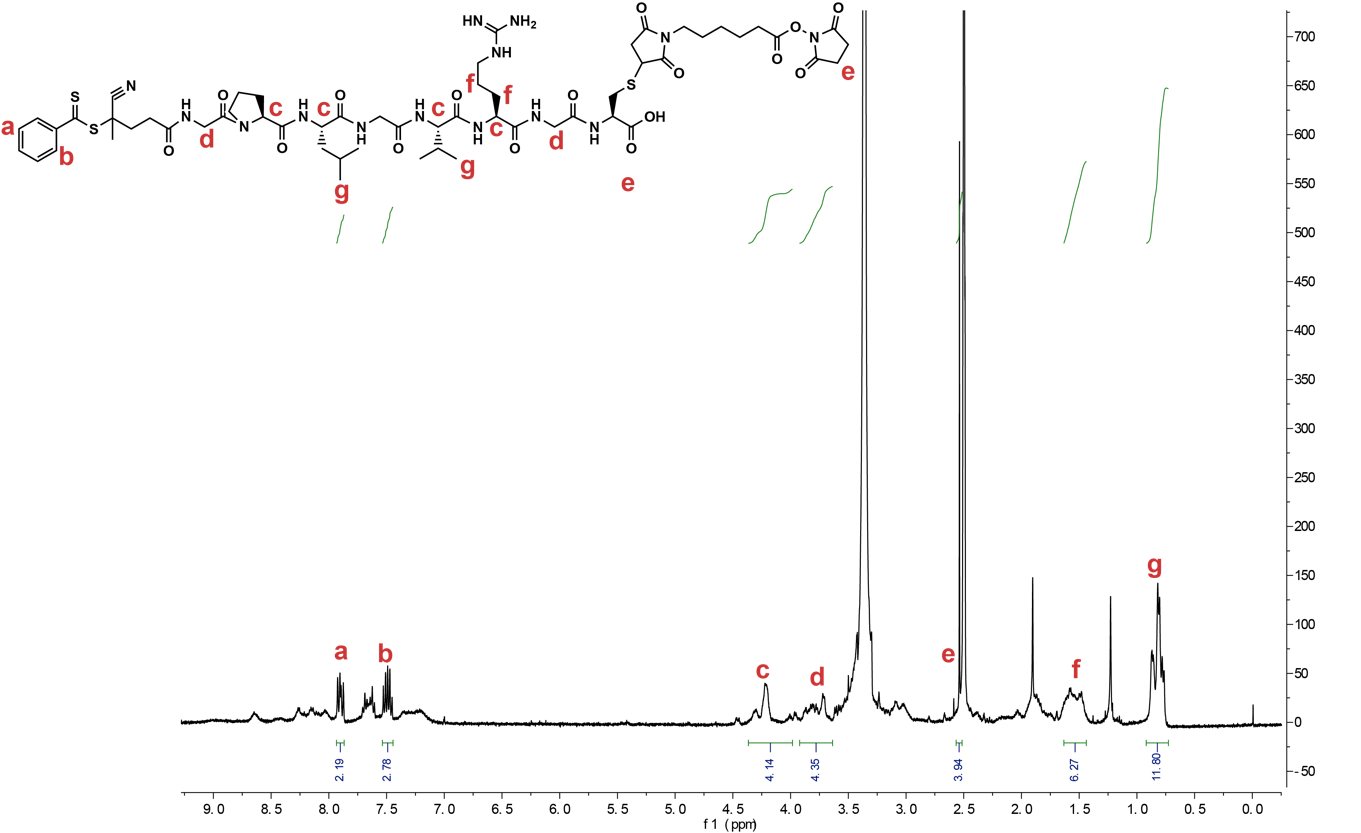


**Figure S4.** The ^1^H NMR spectrum of **RAFT-pep-NHS** in DMSO-d6.


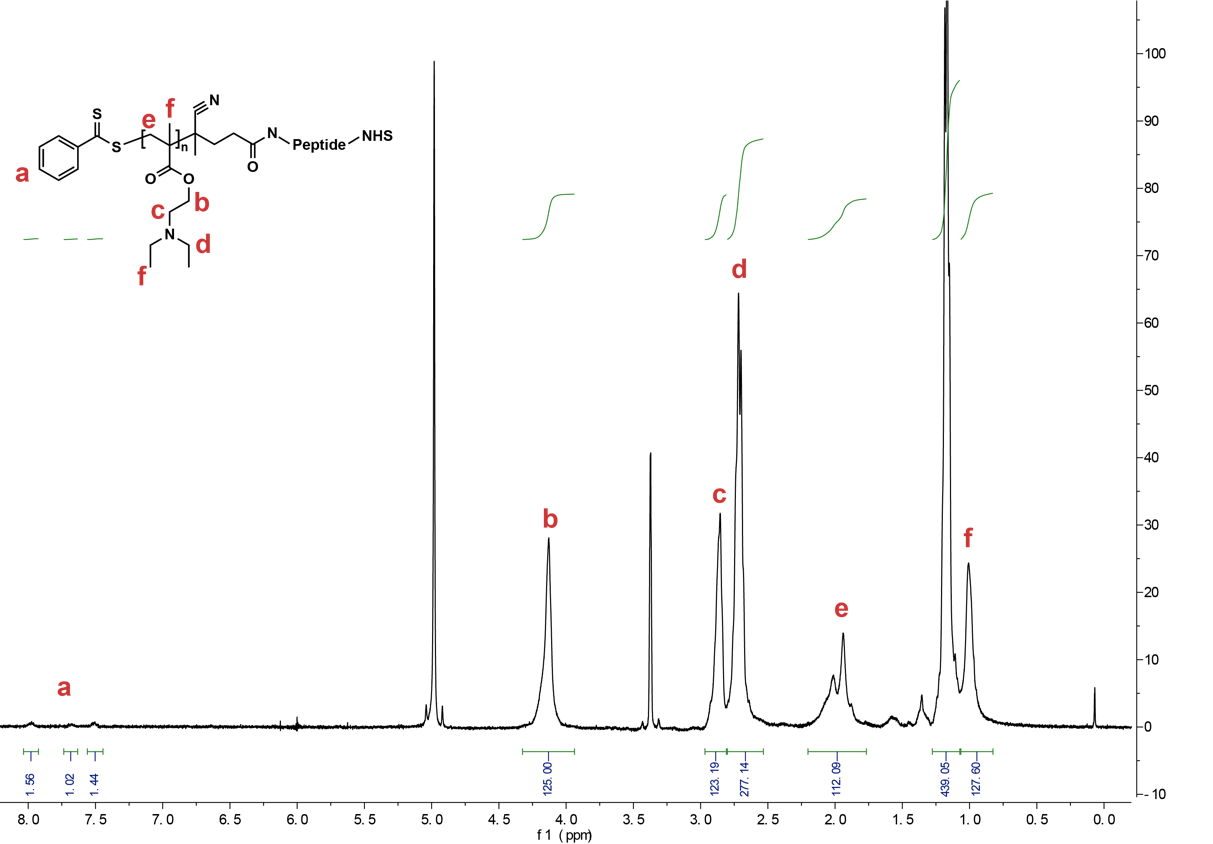


**Figure S5.** The ^1^H NMR spectrum of **PDEA-pep-NHS** in CD_3_OD.


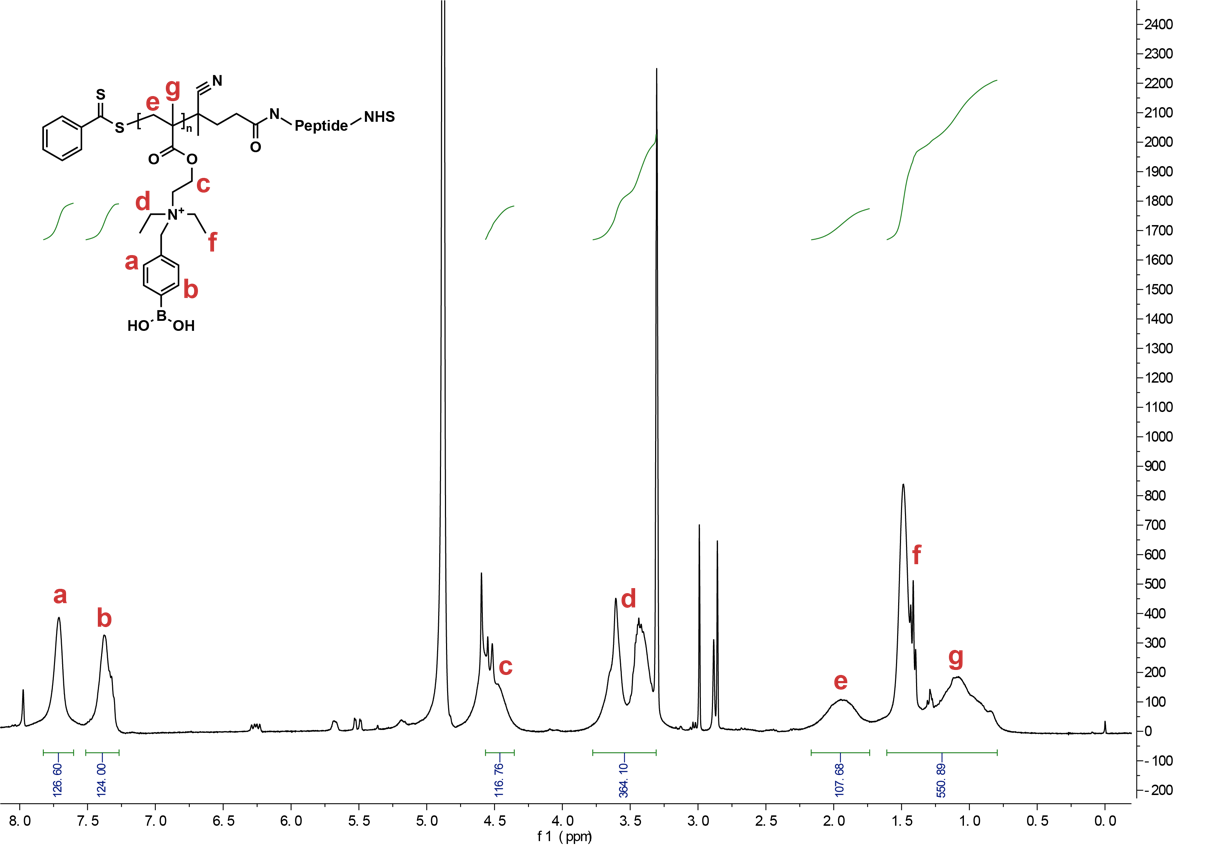


**Figure S6.** The ^1^H NMR spectrum of **B-PDEA-pep-NHS** in CD_3_OD.


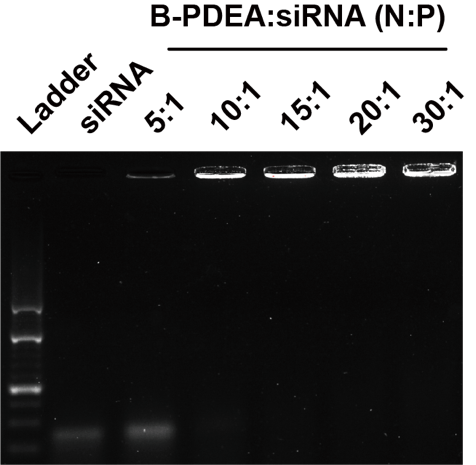


**Figure S7.** Agarose gel electrophoresis of polyplexes at different N/P ratios.


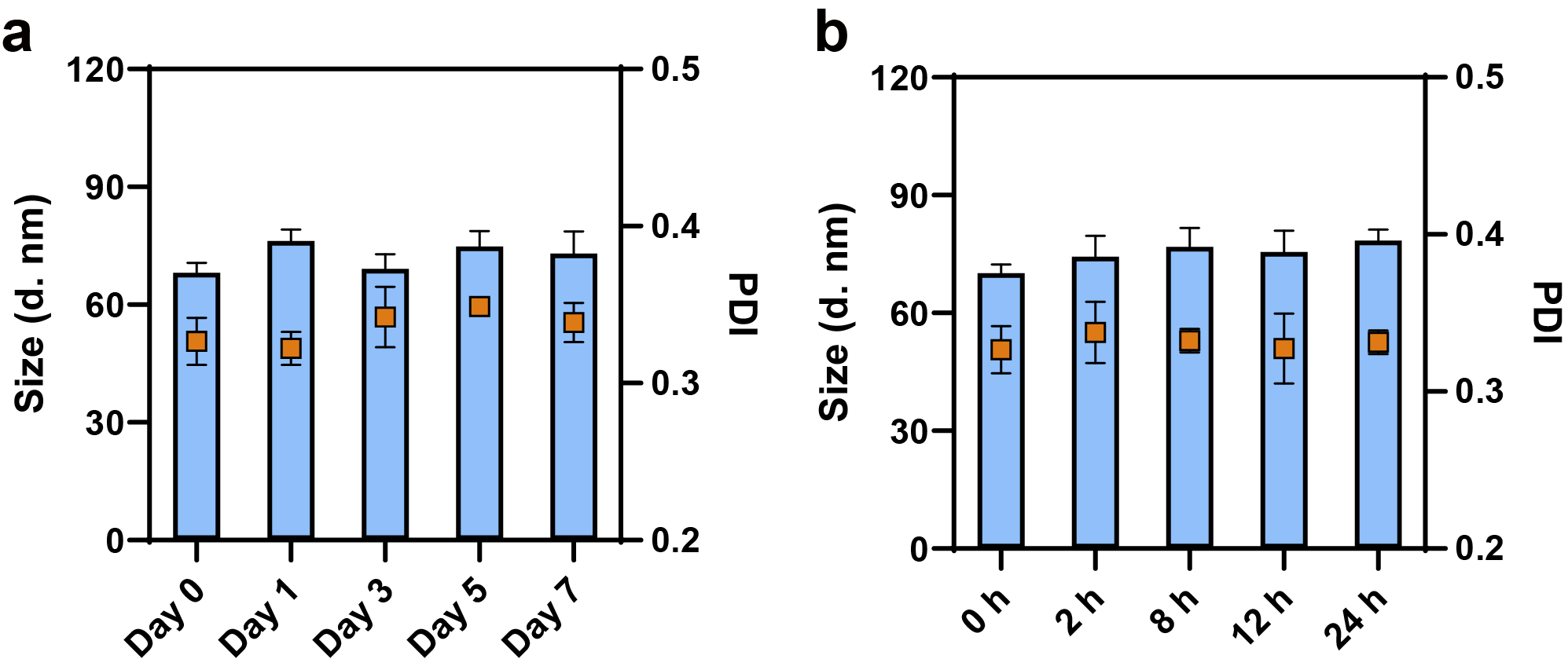


**Figure S8.** Size and PDI changes of B-PDEA@CPA nanoparticles upon incubating with a) PBS (pH 7.4) and b) PBS contain 10% FBS. The data are represented as means ± SD (n = 3).


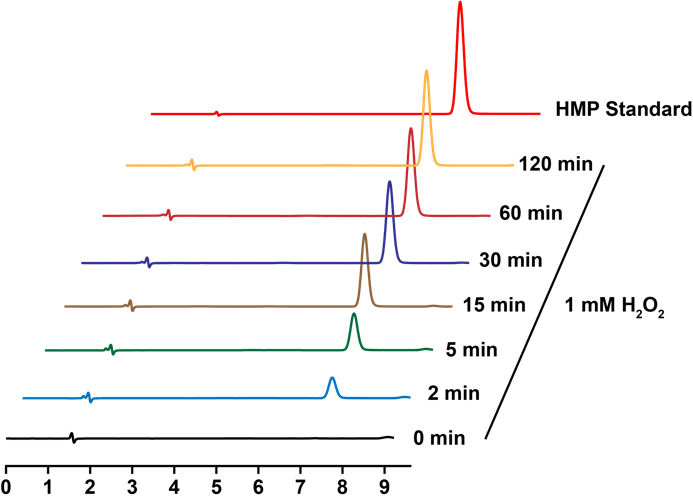


**Figure S9.** The HMP release traced by HPLC of **B-PDEA** incubated with 1 mM H_2_O_2_ at 37 °C.


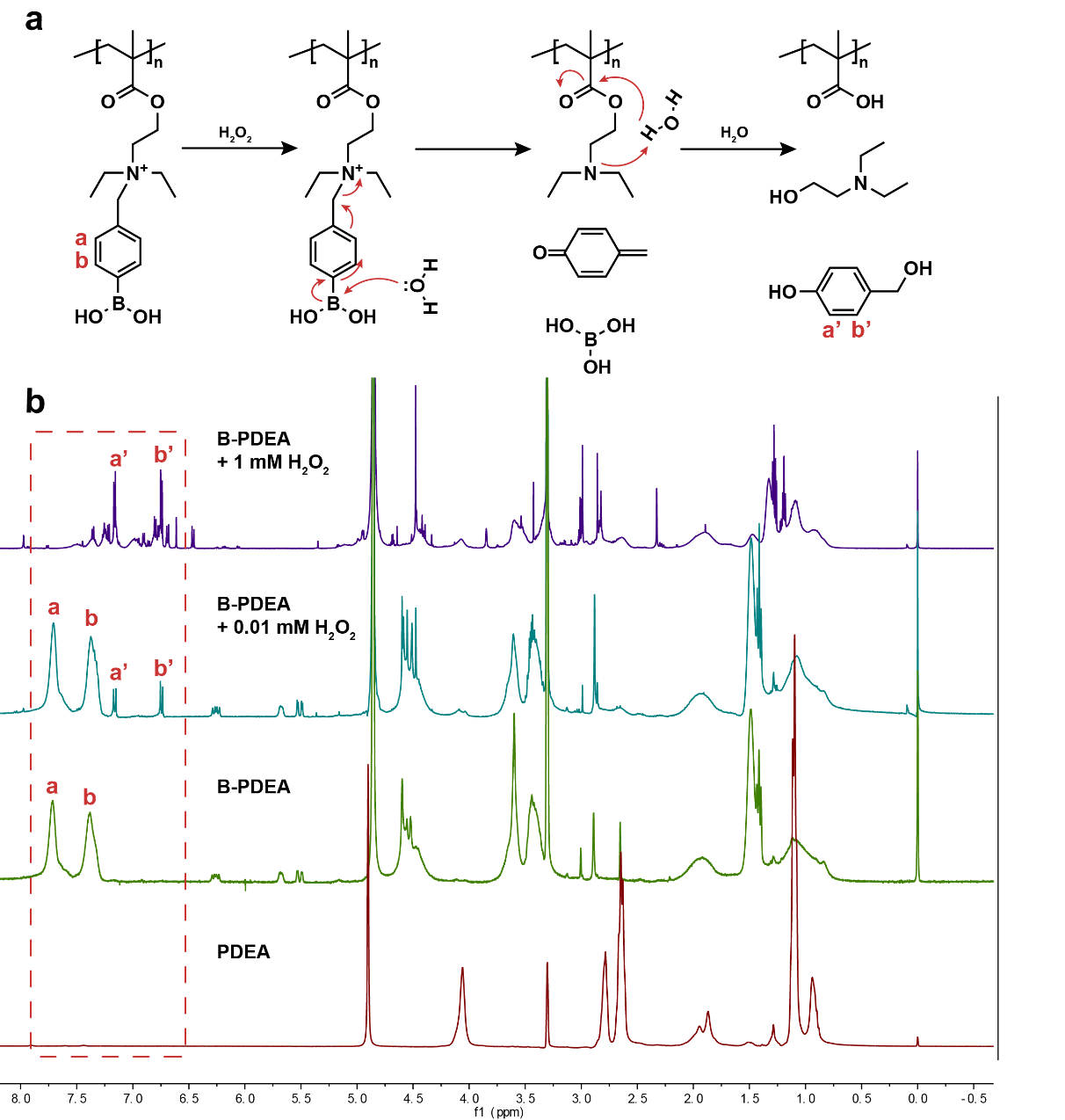


**Figure S10.** The ROS-responsive profile of **B-PDEA**. a) Scheme of the ROS-triggered charge reversal of **B-PDEA**. b) ^1^H-NMR spectra of **B-PDEA** after treated with different concentration of H_2_O_2_ and self-catalyzed hydrolysis in D_2_O.


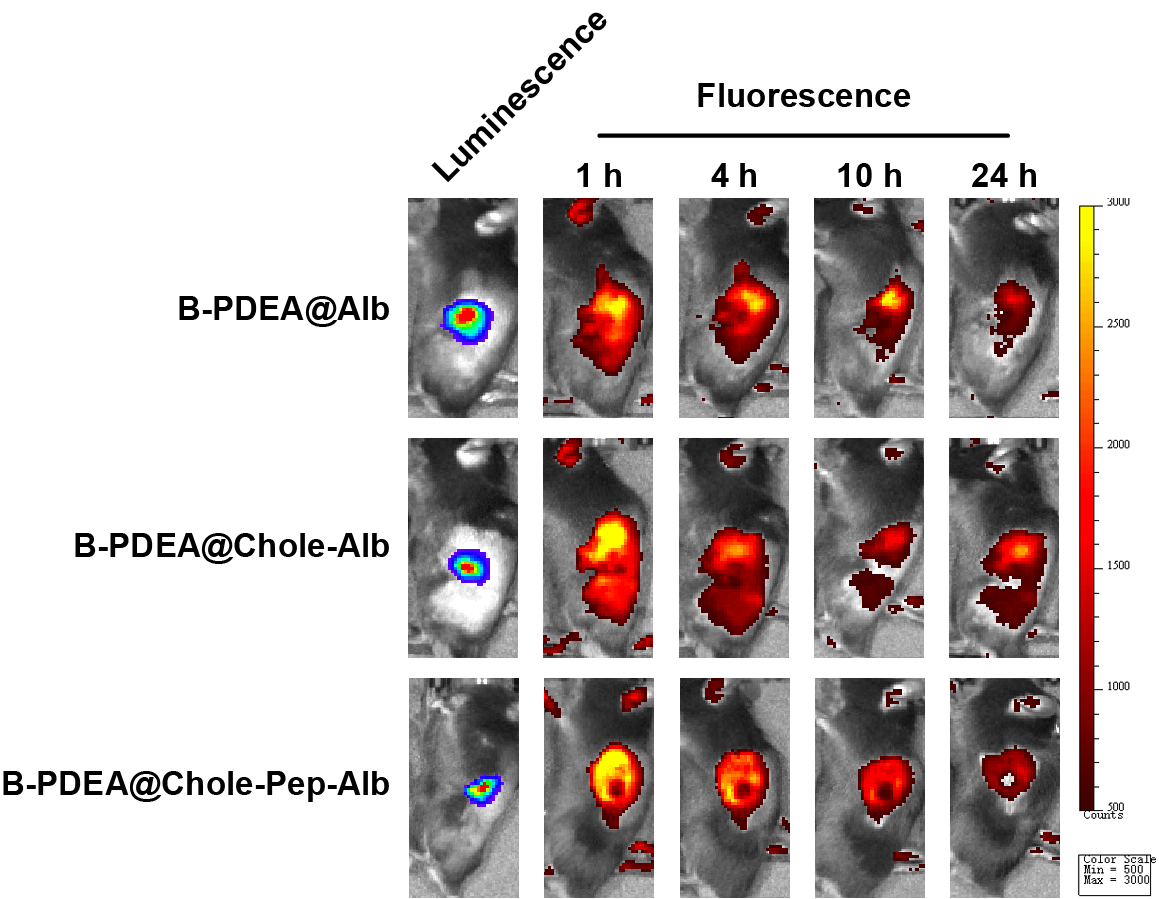


**Figure S11.** *In vivo* luminescence and fluorescent images of B-PDEA@Alb, B-PDEA@Chole-Alb and B-PDEA@Chole-Pep-Alb post 1, 4, 10, and 24 h i.v. injection.


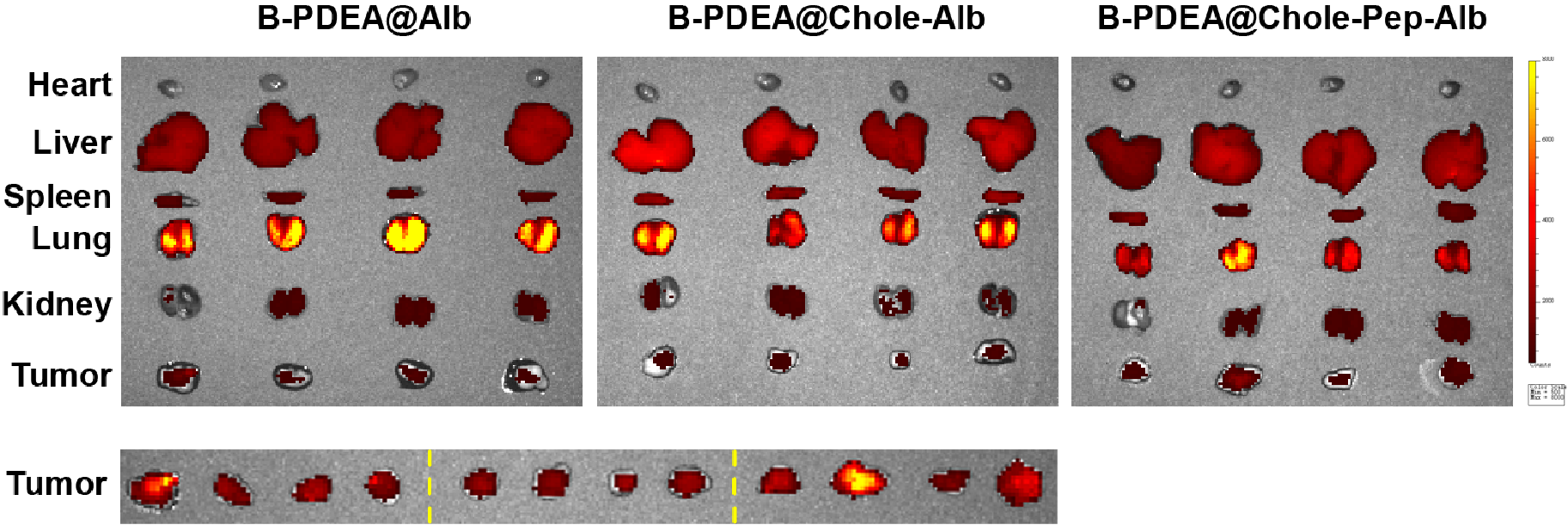


**Figure S12.** Biodistribution of B-PDEA@Alb, B-PDEA@Chole-Alb and B-PDEA@Chole-Pep-Alb post 24 h i.v. injection in main organs and tumors.


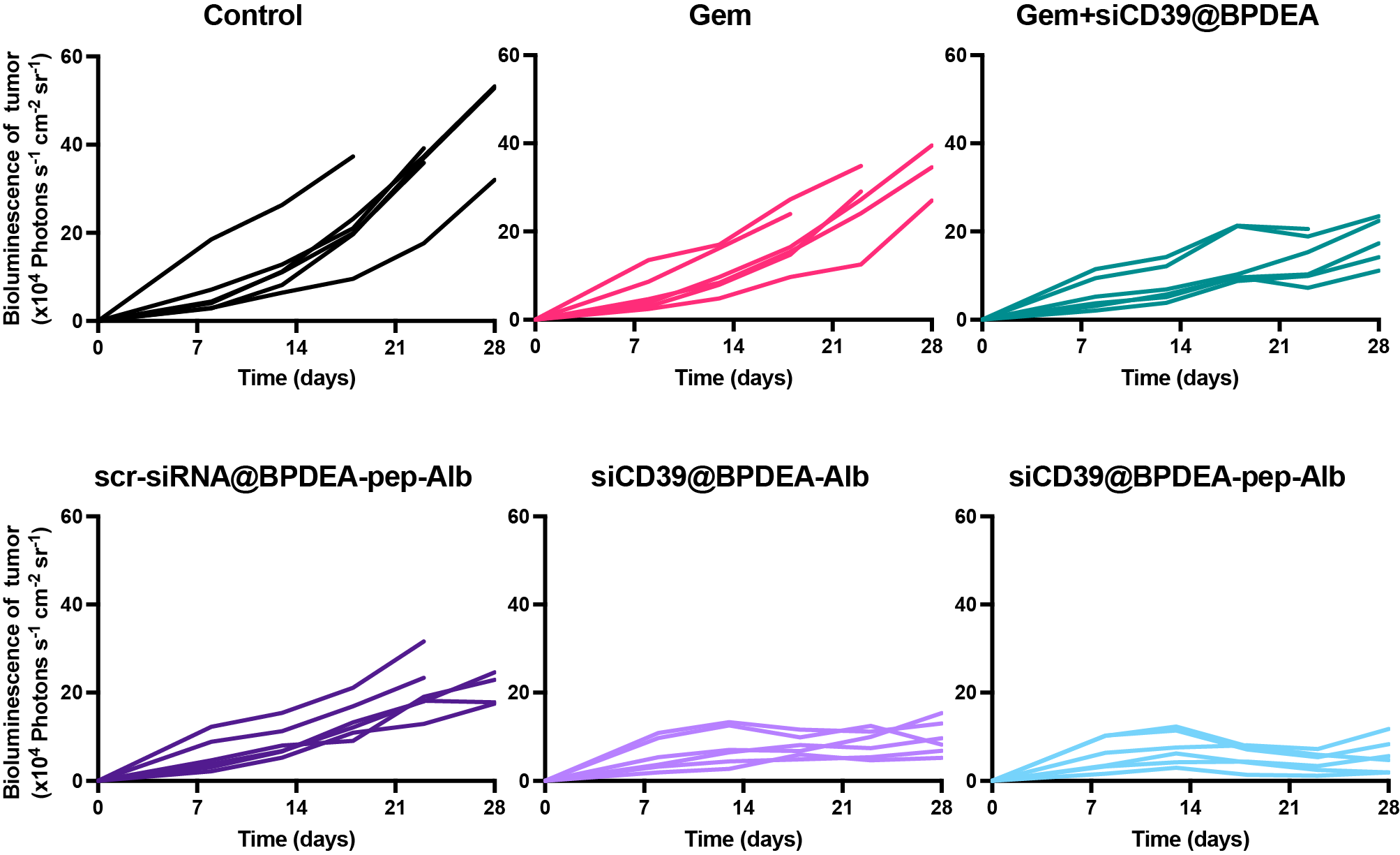


**Figure S13.** Tumor volume changes of each treating group measured by the bioluminescence signal.


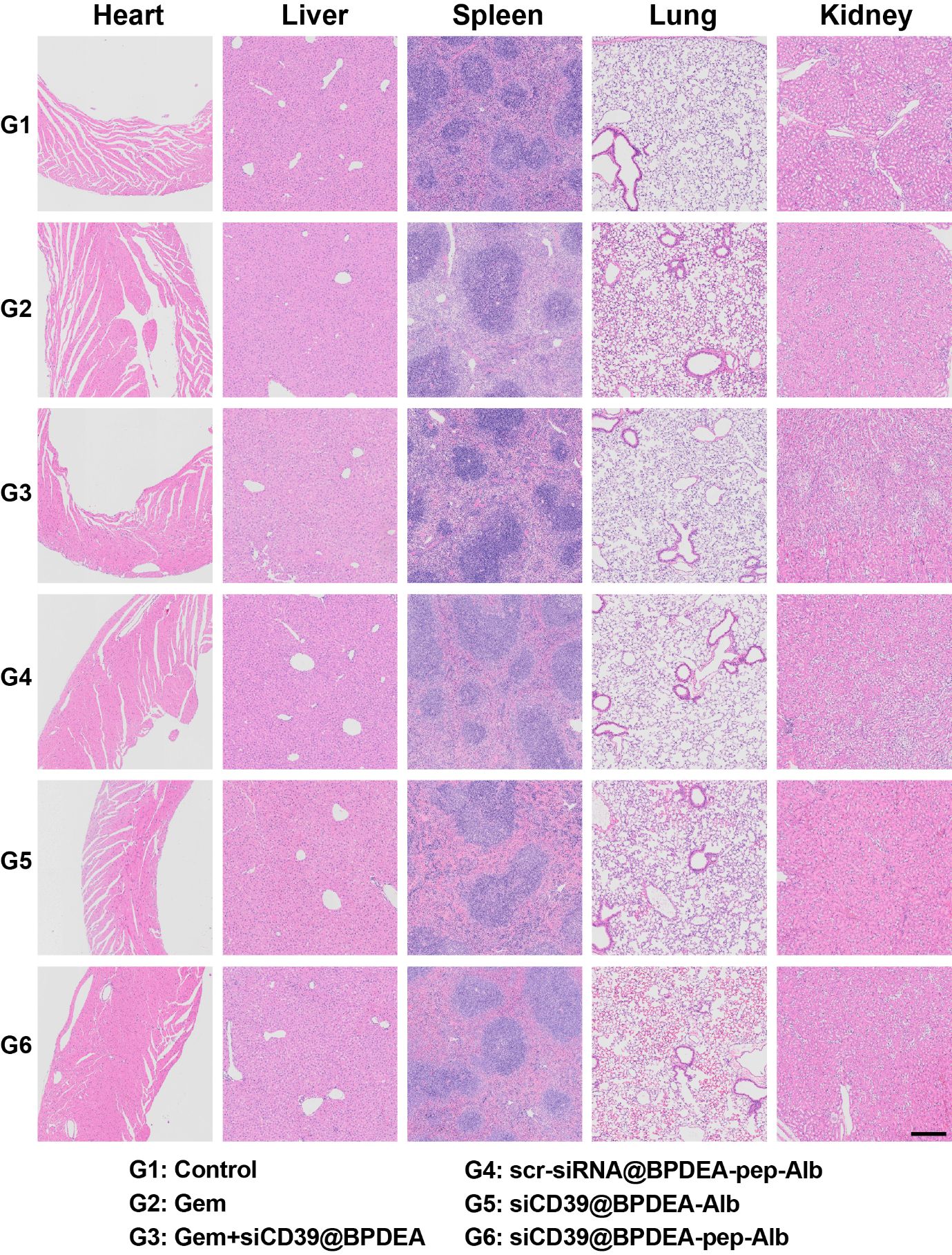


**Figure S14.** Representative images of H&E staining of major organs (heart, liver, spleen, lung, and spleen) from KPC xenograft mice treated with different formulations of Day 20^th^. Scale bar = 500 μm.


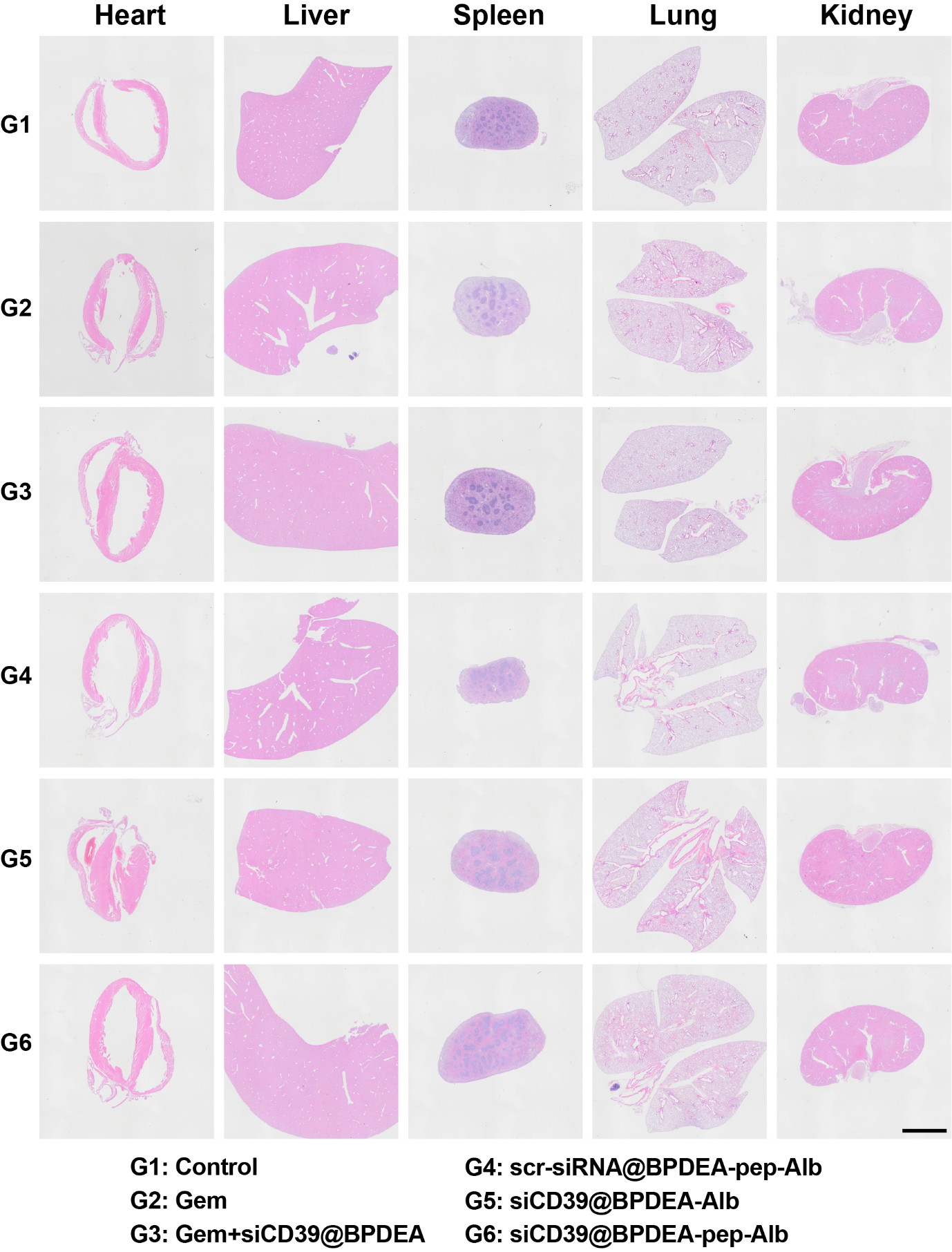


**Figure S15.** Slide scan images of H&E staining of major organs (heart, liver, spleen, lung, and spleen) from KPC xenograft mice treated with different formulations of Day 20^th^. Scale bar = 2 mm.


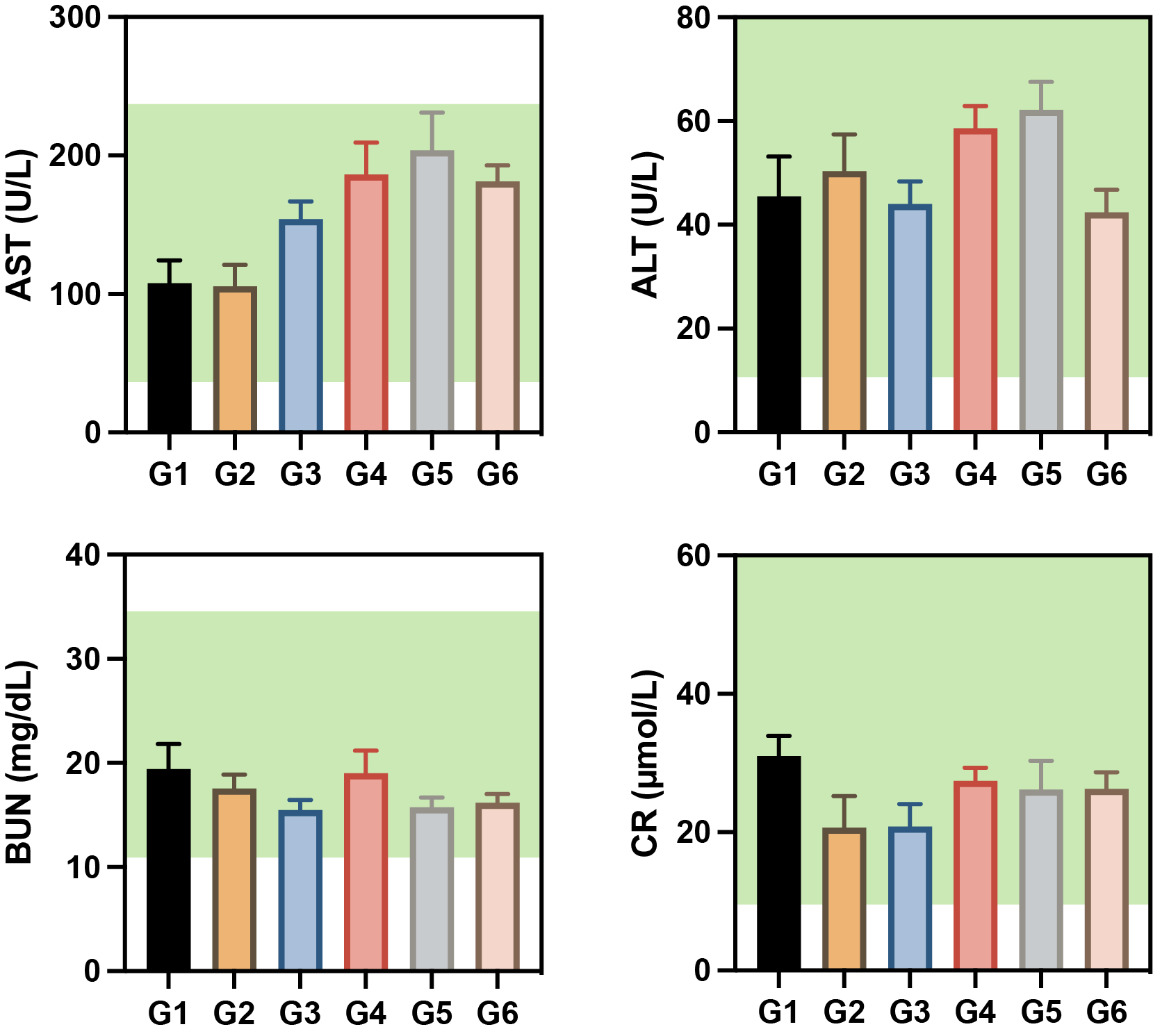


**Figure S16.** Liver enzyme levels (AST and ALT) and kidney function indicators (BUN and CR) in mice treated with different formulations of Day 20^th^. The data are represented as the mean ± SD (n = 3). The green shading indicates the normal ranges of each indicator. AST, aspartate aminotransferase; ALT, alanine aminotransferase; BUN, blood urea nitrogen; CR, serum creatinine.


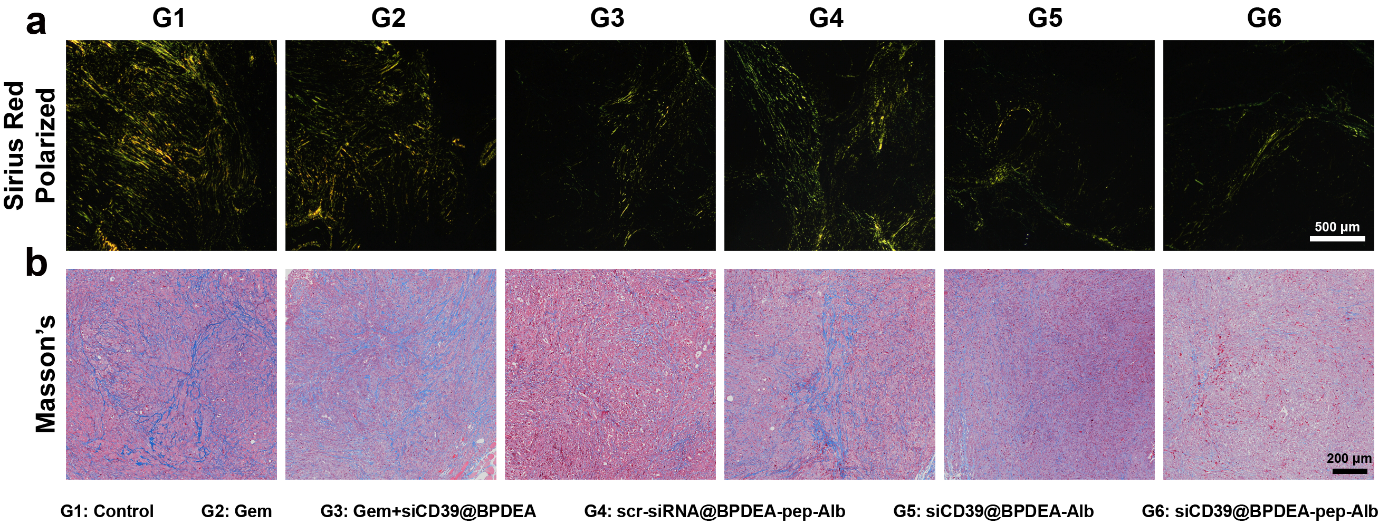


**Figure S17.** a) Representative polarized images of Sirius Red staining indicating collagen in tumor tissues. Scale bar = 500 μm. b) Representative images of Masson’s trichrome staining indicating collagen in tumor tissues. Scale bar = 200 μm.


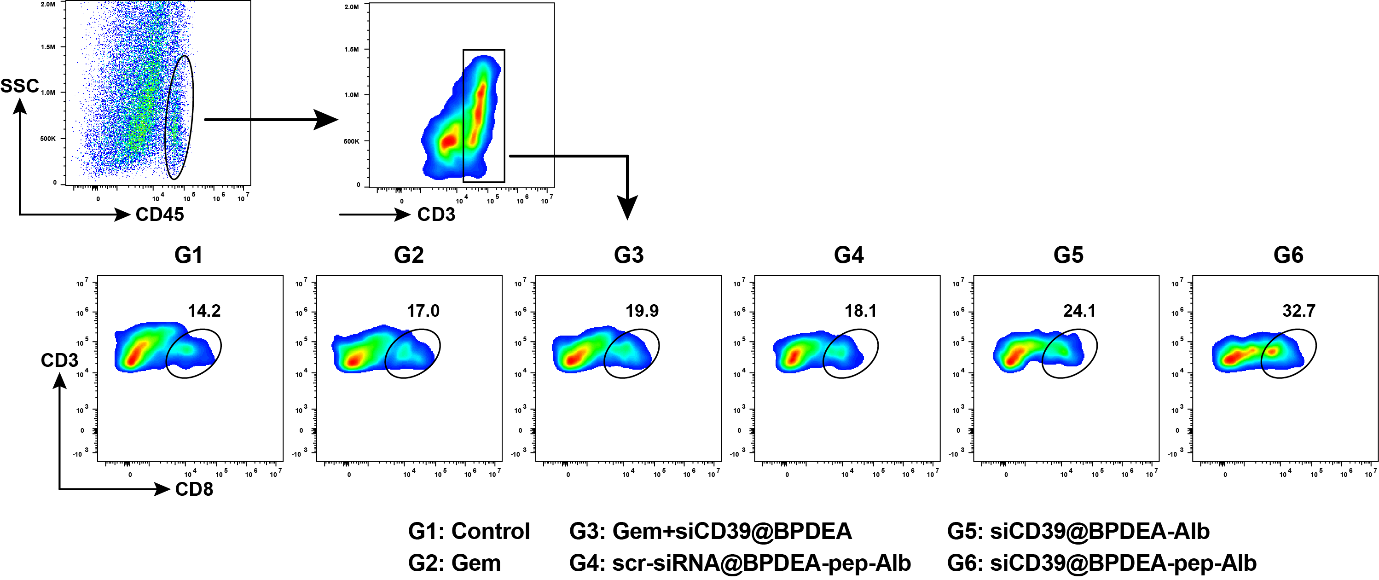


**Figure S18.** Gating strategy to determine frequencies of CD8^+^ T cells. Representative FACS analysis plots of cytotoxic T cells (CD45^+^CD8^+^) infiltrated in orthotopic PDAC tumors.


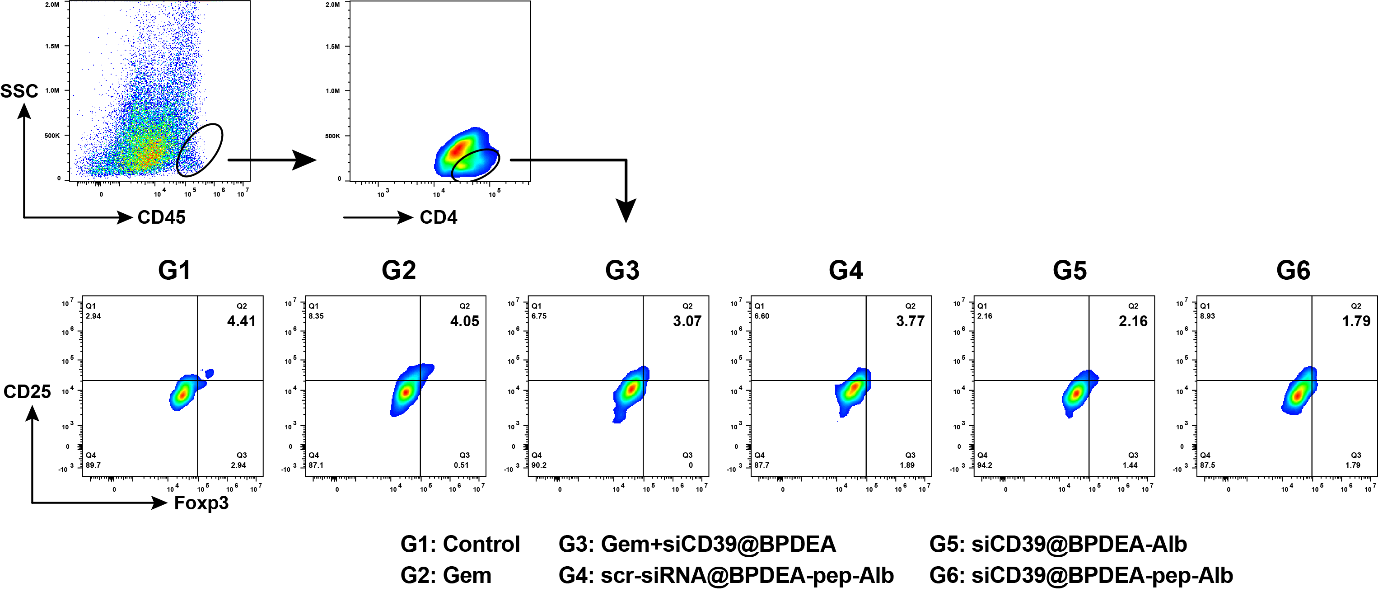


**Figure S19.** Gating strategy to determine frequencies of regulatory T cells (Tregs). Representative FACS analysis plots of Tregs (CD45^+^CD4^+^CD25^+^Foxp3^+^) infiltrated in orthotopic PDAC tumors.


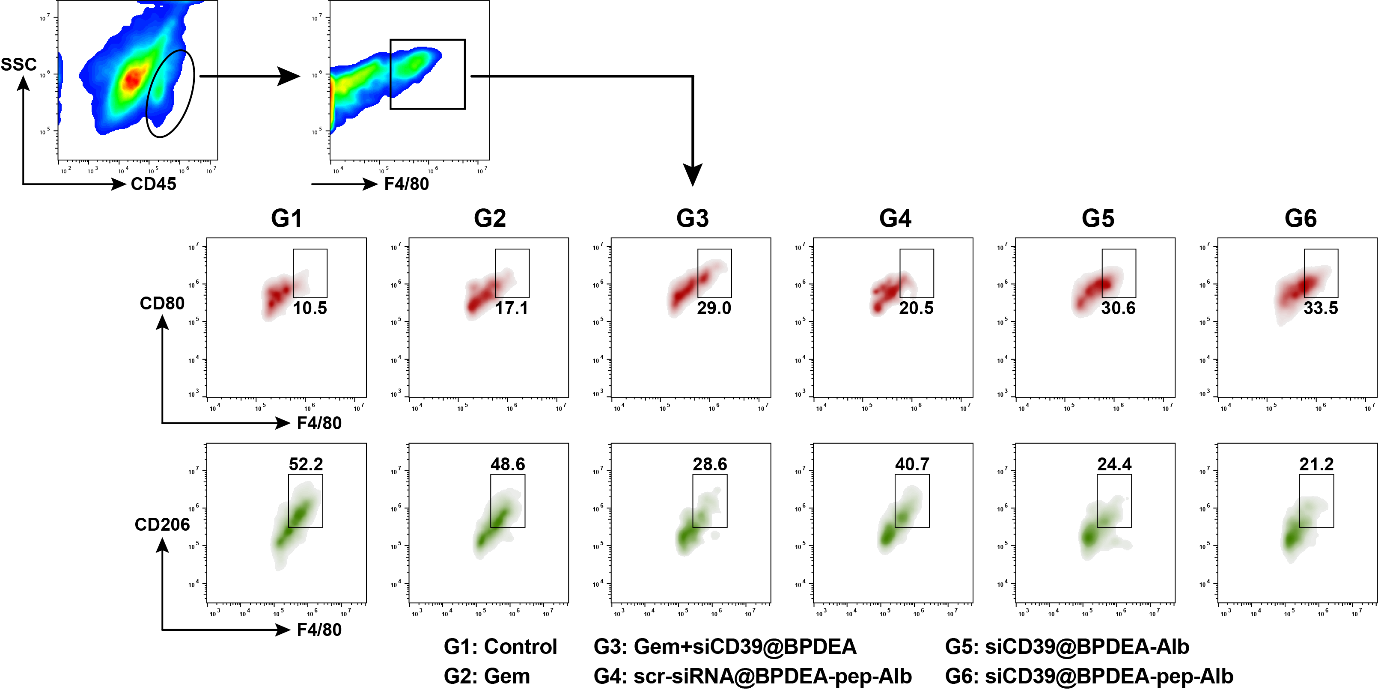


**Figure S20.** Gating strategy to determine frequencies of M1/M2 macrophages. Representative FACS analysis plots of M1 macrophages (CD45^+^F4/80^+^CD80^+^) and M2 macrophages (CD45^+^F4/80^+^CD206^+^) infiltrated in orthotopic PDAC tumors.


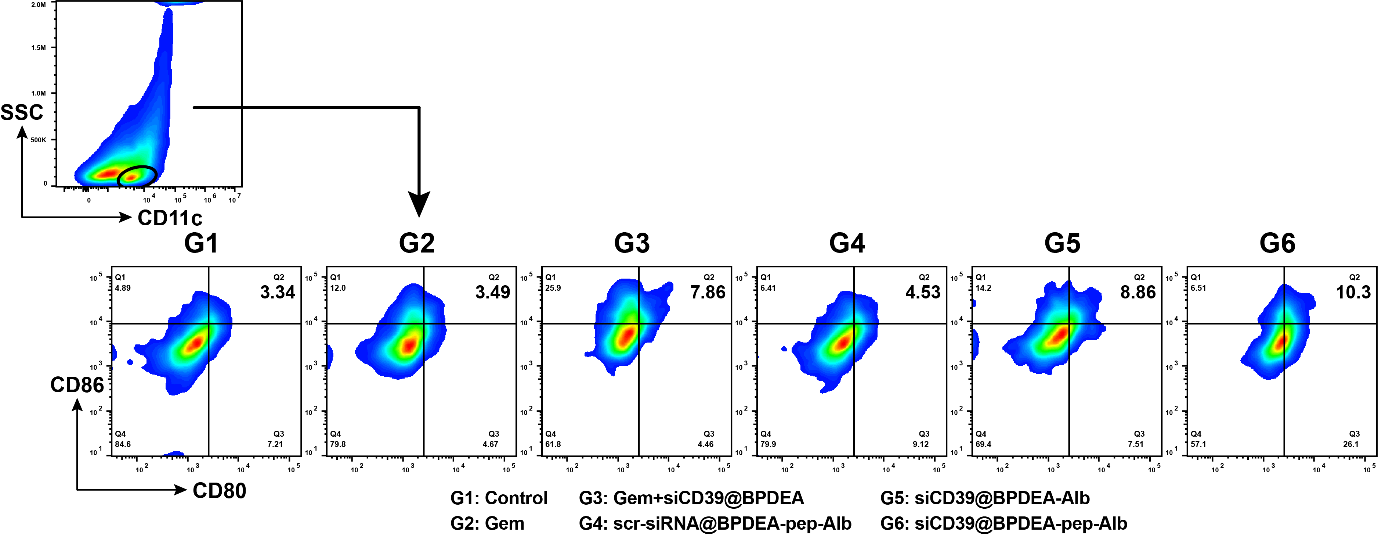


**Figure S21.** Gating strategy to determine frequencies of mature DCs. Representative FACS analysis plots of mature DCs (CD11c^+^CD80^+^CD86^+^) in tumor-draining lymph nodes.
